# Dietary choice for a balanced nutrient intake increases the mean and reduces the variance in the reproductive performance of male and female cockroaches

**DOI:** 10.1002/ece3.2243

**Published:** 2016-06-12

**Authors:** Harriet Bunning, Lee Bassett, Christina Clowser, James Rapkin, Kim Jensen, Clarissa M. House, Catharine R. Archer, John Hunt

**Affiliations:** ^1^ Centre for Ecology and Conservation College of Life and Environmental Sciences University of Exeter Tremough Campus Penryn TR10 9EZ UK; ^2^ Department of Entomology North Carolina State University Gardner Hall Raleigh North Carolina 27695‐7613; ^3^ MaxNetAging School Max Planck Institute for Demographic Research Konrad‐Zuse‐Straße 1 18057 Rostock Germany

**Keywords:** Geometric framework of nutrition, optimal foraging, pheromones, sexual selection

## Abstract

Sexual selection may cause dietary requirements for reproduction to diverge across the sexes and promote the evolution of different foraging strategies in males and females. However, our understanding of how the sexes regulate their nutrition and the effects that this has on sex‐specific fitness is limited. We quantified how protein (P) and carbohydrate (C) intakes affect reproductive traits in male (pheromone expression) and female (clutch size and gestation time) cockroaches (*Nauphoeta cinerea*). We then determined how the sexes regulate their intake of nutrients when restricted to a single diet and when given dietary choice and how this affected expression of these important reproductive traits. Pheromone levels that improve male attractiveness, female clutch size and gestation time all peaked at a high daily intake of P:C in a 1:8 ratio. This is surprising because female insects typically require more P than males to maximize reproduction. The relatively low P requirement of females may reflect the action of cockroach endosymbionts that help recycle stored nitrogen for protein synthesis. When constrained to a single diet, both sexes prioritized regulating their daily intake of P over C, although this prioritization was stronger in females than males. When given the choice between diets, both sexes actively regulated their intake of nutrients at a 1:4.8 P:C ratio. The P:C ratio did not overlap exactly with the intake of nutrients that optimized reproductive trait expression. Despite this, cockroaches of both sexes that were given dietary choice generally improved the mean and reduced the variance in all reproductive traits we measured relative to animals fed a single diet from the diet choice pair. This pattern was not as strong when compared to the single best diet in our geometric array, suggesting that the relationship between nutrient balancing and reproduction is complex in this species.

## Introduction

Optimal foraging theory predicts that animals regulate their nutrient intake to maximize their fitness (Stephens and Krebs 1986). While traditional optimal foraging models assumed that fitness maximization relied on high energy intake (Stephens and Krebs 1986), recent work shows that foragers must regulate both how much energy they eat and also the nutrients that this energy comes from (Simpson and Raubenheimer 2012). This is because expressing fitness determining traits depends on the amount and combination of nutrients eaten. For example, in fruit flies (*Drosophila melanogaster* – Lee et al. 2008; Jensen et al. 2015a), egg laying rate is maximized in females that eat energy‐rich food containing protein (P) and carbohydrate (C) in a 1:2 ratio, while life span is best when females eat a 1:16 ratio of P:C. These trait‐specific dietary optima mean that the best diet for overall fitness depends on which traits individuals must invest in (e.g., development, somatic maintenance, reproduction), given their age, condition, and environment (Lihoreau et al. 2015; Senior et al. 2015).

Dietary optima may differ considerably across the sexes, especially if sexual selection causes the nutrients needed for reproduction to diverge between males and females. Sexual selection is most pronounced in species where one sex (usually females) invests more intensely in offspring than the other sex (usually males) (Trivers 1972). While females allocate resources toward producing offspring, males instead invest in mating with as many females as possible. Sexual selection promotes the evolution of traits that help males attract females or outcompete other males in the population. Expressing these sexually selected traits may require specific nutrients or minerals, such as carotenoids to pigment plumage (Walker et al. 2014) or minerals to make antlers (Landete‐Castillejos et al. 2007). Consequently, sexual selection may drive the evolution of sex‐specific nutritional optima for reproduction (Maklakov et al. 2008). In support of this view, in the field cricket (*Gryllus veletis*), energy determines how intensely males sing to attract females, while females need to eat protein‐rich diets to maximize egg laying (Harrison et al. 2014). Similarly, in Australian field crickets (*Teleogryllus commodus*), protein intake is vital for female egg laying but has a minimal influence on male calling effort (Maklakov et al. 2008). In *D. melanogaster,* males maximize their offspring production rate when they consume large volumes of carbohydrate‐rich diets (P:C = 1:16) and females maximize egg laying rate when they consume a 1:2 P:C ratio (Jensen et al. 2015a). These results suggest that sex‐specific effects of nutrition on reproductive performance are likely to be widespread but we lack data quantifying how nutrition affects reproduction and overall fitness in the sexes. Moreover, we have a poor understanding of how males and females regulate their nutrient intake how nutrient balancing affects their reproductive performance.

Foragers can regulate their nutrient intake in several ways (Simpson and Raubenheimer 2012). The simplest way is to adjust how much they eat of a single food. In many species, if an individual is restricted to a single diet that is deficient in an important nutrient, they eat larger volumes of that diet, a process known as compensatory feeding (Raubenheimer and Simpson 1993). Compensatory feeding has been shown in whitebellied sunbirds that adjust their consumption to maintain constant energy intake when fed a range of sucrose solutions (0.07–2.5_M_) (Nicolson and Fleming 2003). If the only food available is nutritionally imbalanced (i.e., contains a mix of nutrients that is suboptimal for fitness), then an individual must decide how much of that food to consume given the costs of over‐ and under‐ingesting nutrients in that food. The geometric framework of nutrition allows us to identify how foragers resolve this trade‐off and prioritize their intake of different nutrients (Simpson and Raubenheimer 2012). Following this approach, a geometric array of diets is constructed that vary in the ratio and total concentration of nutrients they contain. In the simplest case, this is a two‐dimensional array where each axis represents a different nutrient (e.g., Figure S1). Diets in this array are distributed across nutritional rails, whereby diets on each rail contain the same ratio of nutrients, but the total nutritional content of the diet increases as it moves further away from the origin. When restricted to a single diet, individuals can only feed along a single rail by eating more or less of the diet. When different individuals are tested in this manner across all diets in the array, the average intake of nutrients on each nutritional rail can be estimated and the line connecting these values is known as the intake array (Simpson and Raubenheimer 2012). The slope of this intake array provides an indication of how individuals prioritize their intake of nutrients. For example, Jensen et al. (2012) used this approach to show that when female predatory beetles (*Anchomenus dorsalis*) are restricted to diets varying in P and lipid (L) content, they more strongly prioritize their intake of L over P. That is, the slope of the intake array for these nutrients (where P intake is on the *x*‐axis and L intake on the *y*‐axis) was −0.26. This differed significantly from a slope of −1, which would indicate that equal priority is given to both nutrients and also from a slope of −0.5, expected if priority is given to energy intake because L contains twice as much energy as P per gram ingested. Furthermore, the coefficient of variation for L intake across females was approximately 25% lower than for the intake of P. Collectively, this suggests that the costs of over‐ or underingesting L exceed those of under‐ or over‐eating P in *A. dorsalis* (Jensen et al. 2012).

As well as regulating how much they eat, foragers can eat from different nutritionally imbalanced foods to consume an optimal nutrient blend. To identify how foragers do this, we can provide animals with a pair of nutritionally imbalanced diets and measure how much of each diet they consume. If animals eat nonrandomly from each food in the pair, then it suggests active nutrient regulation. There is even better evidence for nutrient regulation if foragers given different pairs of foods adjust their intake to consistently eat the same ratio of nutrients. This nutrient ratio is called the “regulated intake point”, which under optimal nutrient balancing coincides with the “intake target” where fitness is maximized (Simpson and Raubenheimer 2012). This approach has revealed that many species can regulate their nutrient intake around a particular intake point. For example, When offered pairs of foods varying in their P and C content, female fruit flies eat nonrandomly from each food to converge on a P:C ratio of 1:4 (Lee et al. 2008; Jensen et al. 2015a). Similarly, acellular slime molds (Dussutour et al. 2010), crickets (Maklakov et al. 2008; Harrison et al. 2014), and caterpillars (Lee 2010) can all fine‐tune their intake to a specific P:C ratio, while predatory beetles (*A. dorsalis*) regulate their consumption to a specific P:L ratio (Jensen et al. 2012).

In theory, these intake strategies should improve a forager's overall fitness or the expression of particular traits that are closely linked to fitness. In agreement with this prediction, slime molds allowed to regulate their intake choose a P:C ratio that optimizes growth rates (Dussutour et al. 2010) and female predatory beetles given a choice of diets optimize their fecundity (Jensen et al. 2012). However, *D*. *melanogaster* allowed to regulate their intake choose a diet that does not allow maximal rate of offspring production (Lee et al. 2008; Jensen et al. 2015a). This is surprising given that this trait is a key determinant of fitness. Similarly, the few studies that have considered sex differences in nutrient regulation have shown that males and females do not always eat a diet that maximizes their performance. When allowed a choice of foods, female field crickets (*G. veletis*) eat more protein than males to meet the high protein requirements of egg laying (Harrison et al. 2014). However, in *D. melanogaster* (Jensen et al. 2015a) and the cricket *T. commodus* (Maklakov et al. 2008), both sexes eat the same P:C ratio when allowed a choice despite having different dietary optima for reproduction (Maklakov et al. 2008; Jensen et al. 2015a). Therefore, it appears that foragers do not always choose a nutrient blend that is likely to maximize their fitness and it is unclear how often males and females can regulate their diet to reach their own dietary optima.

Even if foragers do not choose a nutrient ratio that improves the expression of a fitness determining trait, simply being able to fine‐tune their dietary intake might have a positive effect on an individual's fitness (Senior et al. 2015). Dietary optima can vary enormously between individuals. For example, in honeybees dietary requirements shift as animals age (Archer et al. 2014; Paoli et al. 2014). In caterpillars, dietary demands differ in immune challenged and healthy animals (Povey et al. 2014), and in grasshoppers, predation risk influences dietary optima (Hawlena and Schmitz 2010). Given these differences in dietary requirements, individuals may have subtly different dietary requirements and need to consume different nutrient blends to increase their fitness (Lefcheck et al. 2013; Lihoreau et al. 2015; Senior et al. 2015). This idea, known as the balanced diet hypothesis (DeMott 1998), has been tested in two recent meta‐analyses that compared the fitness of generalist consumers across trophic levels when constrained to a single diet or when given a choice of diets (Lefcheck et al. 2013; Senior et al. 2015). The first of these studies (Lefcheck et al. 2013) only found weak support for the balanced diet hypothesis showing that although dietary choice increased mean fitness on average, in some cases eating a single best diet provided equal mean fitness. In contrast, a more recent analysis by Senior et al. (2015) found stronger support for this hypothesis showing that, on average, dietary choice increased the mean and reduced the variance of fitness‐related traits relative to individuals consuming a single diet. This pattern became more pronounced as the number of available diets to choose from increased (Senior et al. 2015). These studies strongly suggest that dietary choice has important fitness consequences, however we lack data explicitly testing the effects of dietary choice and nutrient balancing on reproductive traits across the sexes.

Sexual selection in the cockroach, *Naupheota cinerea,* is well documented (e.g., Moore 1990; Moore et al. 1997; Moore and Moore 1999). Male–male competition and female mate choice act contemporaneously in this species and are regulated by the expression of three male sex pheromones: 2‐methylthiazolidine (2MT), 4‐ethyl‐2‐methoxyphenol (4E2M), and 3‐hydroxy‐2‐butanone (3H2B) (Moore and Moore 1999). Females prefer males with high levels of all three pheromones, especially in close range mating trials, whereas higher levels of 3H2B increase the likelihood of a male being subordinate (Moore and Moore 1999). Consequently, the pheromone composition that confers male attractiveness to females and dominance status is opposing (Moore and Moore 1999). Previously, we have shown in this generalist omnivore that both P and C intakes influence sex pheromone expression, with males that consume large amounts of high carbohydrate diets (at a P:C ratio of 1:8) producing high quantities of all three sex pheromones and subsequently being more attractive to females (South et al. 2011). The intake of P and C, however, had little effect on male dominance status (South et al. 2011). We have also shown that males consuming a high intake of nutrients in a P:C ratio of 1:2 produce more sperm and so have higher fertility once they have attracted and mated with a female (Bunning et al. 2015). Thus, males must face a trade‐off between consuming an intake of nutrients that maximizes their attractiveness (1:8 P:C) and one that maximizes their fertility (1:2 P:C). Males appear to resolve this trade‐off by choosing to eat a 1:4.95 P:C ratio, which allows intermediate expression of both traits (Bunning et al. 2015). While the relationship between intake of these nutrients and reproductive performance is well understood in males, we do not know whether P intake and C intake affect female reproductive performance or if females regulate their intake of these nutrients under choice and no‐choice conditions. It is important to note that female *N. cinerea* is ovoviviparous meaning that it produces eggs that are hatched and gestated within a specialized brood pouch of the mother until they hatch as 1st‐instar larvae. As a result, offspring are produced in discrete clutches rather than continuously, which is expected to further magnify the nutritional differences in the sexes.

Here, we use the geometric framework (GF) of nutrition (Simpson and Raubenheimer 2012) to determine the effect of P and C intake on sex‐specific measures of reproductive performance (male sex pheromone expression, female clutch size, and gestation time) in *N. cinerea*. We then determined how each sex regulated their nutrition when restricted to a single diet and when offered a choice between alternate diets. Finally, we determined how nutrient balancing under dietary choice affected reproductive performance in males and females. Our results demonstrate the importance of nutrient balancing to reproductive performance in *N. cinerea*.

## Materials and Methods

### Experimental animals and husbandry

Cockroaches used in this experiment originate from a mass colony of *N. cinerea,* containing over 200,000 individuals. Stock animals are kept in ten plastic containers (50 × 35 × 30 cm) in incubators (28 ± 1°C, 14:10 h L:D). Cultures are fed weekly with a cup of dry rat chow (SDS diets, Essex, UK) and provided with water in two glass vials (15 cm long, 3 cm diameter) plugged with cotton wool. When cultures are cleaned every 2 months, several hundred individuals are transferred between containers to maintain gene flow. This mass culture method prevents inbreeding and preserves substantial levels of genetic variation (Corley et al. 2001).

Experimental cockroaches were removed from cultures as final instar nymphs and housed in single sex cultures in smaller containers (39 × 25 × 6 cm). These animals were supplied with rat chow and water ad libitum. Newly eclosed virgin adults were collected daily from these cultures and allocated randomly to either the no‐choice experiment (Experiment 1), the choice experiment (Experiment 2), or were kept as mating partners for experimental animals.

Cockroaches used in experiments 1 and 2 were housed individually in 17 × 12 × 6 cm plastic containers and provided one (Experiment 1) or two (Experiment 2) diets. Water was provided ad libitum in feeding platforms. Mating partners were housed individually in smaller 11 × 11 × 3 cm plastic containers and provided with rat chow ad libitum and water in small plastic vials (10 cm length, 1.5 cm diameter) plugged with cotton wool. All mating partners were only used once in each experiment to ensure that they were socially naïve and a virgin at the time of mating. As with our mass colony, experimental animals were kept in a constant temperature room at 28 ± 1°C and under a 14:10 L:D lighting regime.

### Artificial diets, feeding regime, and measuring nutrient intake

We constructed 24 powdered, holidic (i.e., chemically defined) diets that varied in their ratio of protein (P) and carbohydrate (C) and their total nutritional content (P + C), using established methods (Simpson and Abisgold 1985). All diets contained P in a 3:1:1 mixture of casein, albumen, and peptone and digestible C in a 1:1 mixture of sucrose and dextrin. To manipulate the ratio of nutrients, different relative amounts of P and C were combined into a mixture. To vary the total nutritional content of diets, these mixtures were diluted to varying degrees with crystalline cellulose, which is indigestible to most insects. Although *N. cinerea* may produce the cellulose digesting enzyme cellulase (Wharton and Wharton 1965), other cellulase‐producing cockroaches (e.g., *Blattella germanica*) have a limited ability to digest cellulose (Jones and Raubenheimer 2001). This means that while cellulose may contribute to the nutrient intake of experimental animals, this contribution is probably minor. All diets contained the same amounts of Wesson's salts (2.5%), ascorbic acid (0.28%), cholesterol (0.55%), linoleic acid (0.55%), and vitamin mix (0.18%). The composition of each diet is provided in Table S1 and the location of diets in nutritional space is illustrated in Fig. S1.

Each experimental animal was weighed on their first day of adulthood using an electronic balance (Ohaus Pro^®^ Explorer Ohaus, Pine Brook, NJ, USA) and randomly assigned to a single diet (Experiment 1) or a pair of diets (Experiment 2) and provided with food of known dry mass. Animals were then fed every 5 days until females gave birth or until males reached 55 days posteclosion (i.e., the average time taken for females to gestate a clutch). Containers were cleaned at each feeding period. Water and food were provided in separate feeding platforms created by gluing the upturned plastic lid of vial (1.6 cm diameter, 1.6 cm deep) in the center of a plastic Petri dish (5.5 cm diameter). The base of these dishes collected any spilt food to provide a more accurate estimate of diet consumption. Diet was dried in an oven at 30°C for 48 h both before being supplied to experimental animals and then again after a given feeding period before any leftover diet was weighed. When leftover food was collected from animals, feces were removed using fine forceps. Diet consumption was calculated as the difference in dry weight before and after feeding and converted into P and C intake by multiplying by the proportion of these nutrients in the diet (Table S1). All experimental animals were mated at 10 days posteclosion to a randomly assigned mating partner, which was also aged 10 days posteclosion. If mating did not occur, another partner was provided until successful mating was observed. Diet was removed from the container during mating and returned immediately after mating. This interval never exceeded more than an hour.

#### Experiment 1: No choice of diets on six nutritional rails at four concentrations

To determine the linear and nonlinear effects of P and C intake on reproductive performance, 10 males and 10 females were assigned to each diet on their day of eclosion (total *n *=* *480 cockroaches). For each female, we measured how many offspring she produced in her first clutch and gestation time (i.e., number of days between mating and parturition). At parturition, each clutch was kept at 4°C for 5 min and offspring were counted. A total of 14 females did not produce any offspring within 70 days after mating due to clutch abortion. The incidence of clutch abortion (scored as 0 = aborted, 1 = not aborted) was unrelated to the linear or nonlinear intake of nutrients (ordinal logistic regression with likelihood ratio test: P: $\chi_{1}^{2}$  = 1.29, *P *=* *0.25; C: $\chi_{1}^{2}$ = 0.07, *P *=* *0.80; P × P: $\chi_{1}^{2}$  = 1.45, *P *=* *0.23; C × C: $\chi_{1}^{2}$  = 2.06, *P *=* *0.18; P × C: $\chi_{1}^{2}$  =* *2.03, *P *=* *0.20). We therefore excluded these females from further analysis.

For each male, we measured the expression of the three sex pheromones (2MT, 4E2M, and 3H2B) using gas chromatography–mass spectrometry (GC‐MS). At 55 days posteclosion, each male was frozen in an individual Eppendorf tube at −80°C for GC‐MS analysis following established protocols. A single male died before this age and was excluded from analysis.

##### Measuring sex pheromones

The sternum of each male was removed by dissection and submerged in 400 *μ*L of HPLC grade dichloromethane containing an internal standard ([E,Z]‐4‐7‐tridecadienyl acetate) at 10 ng/*μ*L in a 1‐mL conical vial and soaked at room temperature for 2 h. The sternum was then removed. An auto‐sampler (Agilent PAL CTC) injected 2 *μ*L of the sample into a DB‐Wax column (30 m × 0.25 mm × 0.25 *μ*m film thickness) in an Agilent 8790 GC coupled with an Agilent 5975 mass spectrometer, using helium as a carrier gas. Inlet temperature was 200°C and the injection was in pulsed split less mode. Following injection, the column temperature was maintained at 50°C for 1.5 min, after which it was raised at a rate of 10°C/min until it reached 250°C where it was held for 2 min. The MS transfer line was set at 240°C. The mass spectrometer was operated in selected ion mode, and output was limited to the three target compounds of interest: 3H2B, 2MET, and 4E2M. Analyses were further limited to ions 45, 88, 103, 79, 107, 137, and 152. Our samples were quantified against a multilevel calibration curve containing the target compounds at known concentrations (South et al. 2011).

##### Statistical analysis

Prior to analysis, it was necessary to modify our data in several ways. First, as the number of days feeding varied between the sexes (i.e., 55 days in males and until the birth of the first clutch in females), we expressed the intake of nutrients per day feeding. Second, because females typically consume more than males and our reproductive traits were measured in different units (i.e., pheromones in *μ*g, gestation time in days and clutch size as a count), we standardized these predictor and response variables to a mean of zero and standard deviation of 1 using a *Z*‐transformation. This transformation was necessary to ensure that any observed differences in our nutritional landscapes were not due to differences in scale. Finally, although rapid gestation has been shown to reduce female life span (Moore et al. 2003), females that have a shorter gestation time have the potential to produce more clutches over their lifetime (Schimpf et al. 2012). Therefore, we view a short gestation as a positive indicator of reproductive performance in females. Consequently, we reversed the sign of standardized gestation time prior to analysis to ensure a more straightforward interpretation when compared to other reproductive traits where high values represent increased reproductive performance. Thus, females with faster gestation had positive *z*‐scores, whereas those with slower gestation had negative *z*‐scores.

We quantified the linear and nonlinear (quadratic and correlational) effects of daily P and C intake on reproductive traits in males and females using a multivariate response surface approach (Lande and Arnold 1983). A full description of this approach is outlined in Text S1 (see also South et al. 2011; Bunning et al. 2015). Nonparametric thin‐plate splines were used to visualize the nutritional landscape for each reproductive trait in the sexes and were constructed using the *Tps* function in the “FIELDS” package of R (version 2.13.0, www.r-project.org). We used the value of the smoothing parameters (*λ*) that minimized the generalized cross‐validation score when fitting each nutritional landscape (Green and Silverman 1993). While all analyses were conducted on the modified data outlined above, nutritional landscapes were constructed using raw values to ease interpretation.

We statistically compared nutritional landscapes using a sequential model building approach (Draper and John 1988) based on partial *F*‐tests (Bowerman and O'Connell 1990). This procedure, as applied to nutritional data, is outlined in detailed in Text S2 (see also South et al. 2011; Bunning et al. 2015). In instances where an overall significant difference was detected, univariate analyses were used to determine which nutrient (P or C) contributed to this effect. This sequential model approach only compares the sign and magnitude of nutritional gradients and does not provide information on directionality. That is, traits may show differences in the sequential model because they are more or less sensitive to the intake of a particular nutrient, even though the optima fall in the same region of the nutritional landscape. We therefore also used trigonometry to calculate the angle (***θ***) between the linear nutritional vectors of the landscapes being compared as:

(1) $$\theta ={\text{cos}}^{-1}\left(\frac{a\cdot b}{\left\|a\|\|b\right\|}\right)$$

where *a* is the linear effects of P and C intake in the first response variable being compared, *b* is the linear effects of these nutrients for the second response variable, $\|a\|=\sqrt{a\cdot a}$ and $\|b\|=\sqrt{b\cdot b}$. If ***θ ***= 0°, the nutritional vectors are perfectly aligned, and the optima for the two reproductive traits reside in the same location in nutrient space. ***θ ***= 180° represents the maximum possible divergence between vectors and indicates that the nutritional optima for the reproductive traits occupy different regions in nutritional space. To determine the significance of ***θ***, we estimated the 95% credible interval (CI) of this angle using a Bayesian approach implemented in the “MCMCglmm” package of R (Hadfield 2010). Full details of the procedure are provided in Text S3.

To examine how males and females resolve this trade‐off balance the costs of over‐ versus underingesting P and C when fed a single diet, we constructed an intake array for each sex. In our experiment, an intake array is the line that connects the average daily intake of P and C by individuals feeding along each of the six nutritional rails (see Fig. 2). The slope of this intake array indicates how individuals prioritize P and C intake. If daily P intake is on the *x*‐axis and daily C intake is on the *y*‐axis, a slope of −1 means that individuals give an equal priority to regulating both nutrients and, because P and C contain equal energy per gram eaten, are regulating toward equal energy intake. If the slope of the intake array is significantly <−1, individuals regulate the intake of C more strongly than P, whereas a slope significantly >−1 indicates they regulate the intake of P more strongly than C. We used an analysis of covariance (ANCOVA) that included sex as a fixed effect, mean daily P intake as a covariate, and the mean daily intake of C as the response variable, to test whether the slope of the intake array differed significantly from a slope of zero and also whether this slope differed significantly across the sexes. The slope of the intake array for each sex (*β*_a_) was tested against a hypothetical slope (*β*_h_) of −1 using a *t*‐test, where (*β*_a_−*β*_h_)/SE_*β*a_ approximates a *t*‐distribution with *n*−2 degrees of freedom. We also calculated the coefficient of variation (CV) for mean daily P and C intake for each sex and compared these within and across the sexes using a *F* ratio test (Zar 1986). We compared CVs rather than variance per se because females, on average, ingest more diet per day than males. A lower CV for the daily intake of a given nutrient indicates that a greater priority is given to regulating this nutrient.

#### Experiment 2: Measuring nutrient intake under dietary choice

To determine how males and females regulate their P and C intake under dietary choice, we provided 20 cockroaches of each sex with one of four pairs of diets (*n *=* *160 cockroaches). Each pair contained one diet with a P:C ratio of 1:8 and one with a P:C ratio of 5:1. These diets are on the outermost rails of the nutritional landscape and so by eating different amounts of each food, cockroaches could eat every possible P:C ratio within the nutritional landscape (Fig. S1). We provided these diets in one of two dilutions (%P+C content), 36% or 84% (Fig. S1). This allows us to see how cockroaches regulate their nutrient intake in response to nutrient dilution. Therefore, we provided four different choices of diets: diet pair 1: 1:8 (36%) versus 5:1 (36%), diet pair 2: 1:8 (84%) versus 5:1 (36%), diet pair 3: 1:8 (36%) versus 5:1 (84%), and diet pair 4: 1:8 (84%) versus 5:1 (84%). Males and females were assigned to each diet pair on their day of eclosion and fed every 5 days for 55 days or, in the case of females, until they gave birth to a clutch of offspring. At the end of these trials, we measured sex pheromones in males and reproductive traits (gestation time and clutch size) in females as outlined for Experiment 1.

##### Statistical analysis

To determine whether male and female cockroaches preferred one diet in each pair over the other, we compared the daily intake of each diet using a paired *t*‐test. To account for differences in the nutrient content of diets in each diet pair, we also compared the difference between the observed intake of P and C from both diets to the expected intake of these nutrients had animals eaten at random. We subtracted the expected intake of P and C from the observed intake of these nutrients for each diet pair and compared this difference to a mean of zero using a one‐sample *t*‐test. Thus, a positive value shows that individuals are ingesting more of a given nutrient than expected if they fed at random and indicates a preference for that nutrient, whereas a negative value shows that individuals are ingesting less of a given nutrient than expected through feeding randomly.

We used a multivariate analysis of variance (MANOVA), including sex, diet pair, and their interaction as fixed effects and the daily P and C intake as response variables to determine how nutrient intake differed across diet pairs and the sexes. Univariate ANOVAs were used to determine which nutrients contributed to any overall multivariate effects and Fisher's least significant difference (LSD) post hoc tests were used to determine which diet pairs differed. We calculated the regulated intake point for males and females, defined as the point in nutrient space that individuals actively defend when given dietary choice (Simpson and Raubenheimer 2012), as the mean daily P and C intake across all diet pairs. Moreover, we used *F* ratio tests to compare the CVs for P and C intake at the regulated intake point in males and females and across the sexes (Zar 1986).

To examine how dietary choice affects reproductive traits in males and females, we analyzed our data in three ways. First, we compared reproductive traits across the diet pairs using separate MANOVAs for each sex, including diet pair as a fixed effect and reproductive traits as the response variables. Within each sex, univariate ANOVAs were used to determine which reproductive traits contributed to any overall multivariate effect and Fisher's LSD post hoc analyses were used to determine which diet pairs (if any) differed significantly. Second, we compared the expression of each reproductive trait in males and females feeding on each of the diet pairs to the expression of the same trait when individuals were fed on either diet in the pair individually. To do this, we (i) calculated the mean expression of reproductive traits from our no‐choice experiment for the two diets presented to cockroaches in each diet pair, (ii) subtracted these mean values from each data point in the choice experiment, and (iii) compared this to a mean difference to zero using a one‐sample *t*‐test. A mean difference of zero is expected if there is no difference in trait expression when there is active choice versus no choice. A positive difference that deviates significantly from a mean of zero indicates that cockroaches express these reproductive traits to a higher degree when exerting dietary choice, whereas a negative difference indicates that cockroaches express these traits to a lesser degree under dietary choice. Finally, using the same approach we compared the *CV* for each reproductive trait in males and females feeding on each of the diet pairs to the *CV* of the same trait when individuals were fed on each diet in the pair individually using an *F* ratio test. In both the comparison of the means and *CV*s for reproductive traits between choice and no‐choice experiments, we use our data twice for each diet pair. To be conservative, we therefore Bonferonni corrected our analyses by using *α *= 0.025.

To determine whether dietary choice increases the mean of reproductive traits relative to the consumption of a single best diet, we analyzed our data in two ways. First, we used ANOVA and Fisher's LSD post hoc analyses to determine which diet from our geometric array maximized mean reproductive trait expression in our no‐choice experiment. Diet 23 (P:C 1:8) was shown to maximize (or minimize, in the case of gestation time) the mean of all reproductive traits (Figure S3) and we therefore refer to this as the single best diet for male and female *N. cinerea*. We then used a one‐sample *t*‐test to compare the reproductive traits of individuals feeding exclusively on this single best diet to the mean reproductive trait taken across diet pairs in our choice experiment. Second, we determined the percentage of individuals in our no‐choice experiment that exceeded the mean reproductive trait taken across diet pairs in our choice experiment. As this approach takes into account all diets used in our no‐choice experiment, it provides a more conservative test of whether individuals restricted to a single diet are able to maximize reproductive trait expression relative to those given dietary choice and consuming a mixed diet.

## Results

### Experiment 1: How does protein and carbohydrate intake affect reproductive traits within and between the sexes?

**Figure 1:**
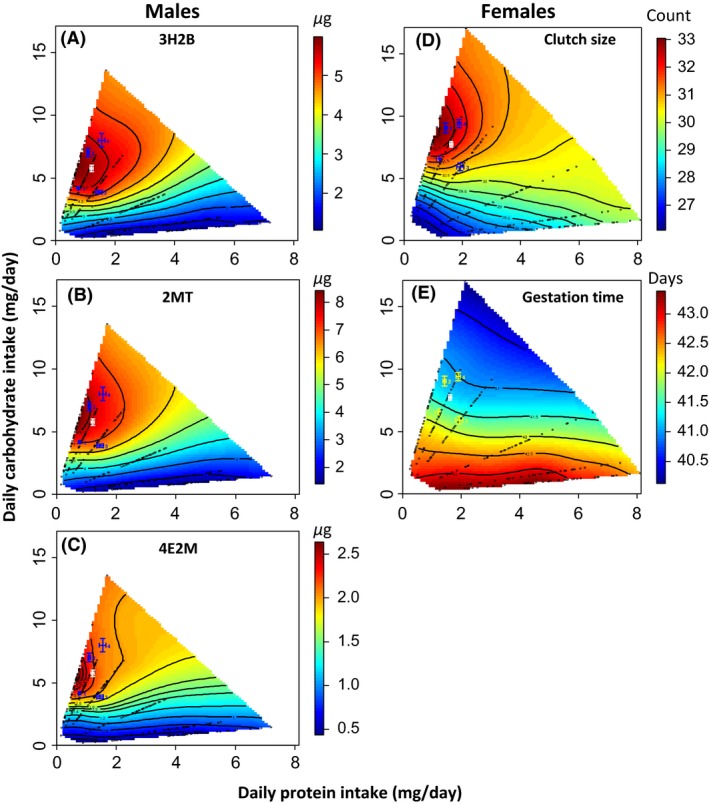
Nutritional landscapes illustrating the effects of daily protein (P) and carbohydrate (C) intake on the expression of the three male sex pheromones, (A) 3H2B, (B) 2MT, and (C) 4E2M, and (D) clutch size and (E) gestation time in females. High values of these reproductive traits are given in red and low values in blue. Black dots represent the intake of these nutrients by individual cockroaches. The blue crosses (yellow in the case of gestation time) on each landscape represent the mean (±SE) intake of nutrients in each of the diet pairs, with the number corresponding to the specific diet pair. The white crosses on each landscape represent the regulated intake point (±SE), calculated as the mean daily intake of P and C across diet pairs.

In males, levels of all three sex pheromones increased linearly with daily C intake but decreased with daily P intake (Table 1). Pheromone expression peaked at daily intake of approximately 6 mg of C, but there were no significant peaks associated with daily P intake (Fig. 1A–C; Table 1). Negative correlational gradients for all three sex pheromones indicate that pheromone expression is maximized on low P and high C diets (1:8 P:C), but this gradient was only statistically significant for 4E2M expression (Fig. 1C; Table 1). Comparing nutrient landscapes for male pheromones using the sequential model building approach revealed no significant differences in the linear, quadratic, or correlational effects of daily P and C intake on pheromone expression (Table 2). Furthermore, the linear nutritional vectors for each pheromone were separated by small angles (<12°). Taken together, these results show that all three pheromones peak in the same high C, low P region of the nutrient landscape (Fig. 1A–C, Table 2).

**Table 1:** The linear and nonlinear effects of daily protein (P) and carbohydrate (C) intake on (A) male sex pheromone expression (3H2B, 2MT, 4E2M) and (B) female clutch size and gestation time

Trait	Linear effects		Nonlinear effects		
	P	C	P × P	C × C	P × C
(A) Males					
3H2B					
Coefficient^a^ ± SE	−0.243 ± 0.054	0.539 ± 0.054	0.024 ± 0.049	−0.172 ± 0.027	−0.131 ± 0.104
*t*_239_	4.546	10.068	0.482	6.279	1.265
*P*	0.0001	0.0001	0.630	0.0001	0.207
2MT					
Coefficient ± SE	−0.227 ± 0.053	0.557 ± 0.053	0.030 ± 0.049	−0.170 ± 0.027	−0.188 ± 0.102
*t*_239_	4.278	10.484	0.608	6.289	1.834
*P*	0.0001	0.0001	0.544	0.0001	0.068
4E2M					
Coefficient ± SE	−0.131 ± 0.061	0.495 ± 0.061	0.011 ± 0.058	−0.149 ± 0.032	−0.254 ± 0.121
*t*_239_	2.163	8.161	0.197	4.670	2.097
*P*	0.032	0.0001	0.844	0.0001	0.037
(B) Females					
Clutch size					
Coefficient ± SE	0.105 ± 0.070	0.351 ± 0.070	−0.079 ± 0.073	−0.142 ± 0.044	−0.356 ± 0.113
*t*_226_	1.489	5.004	1.082	3.263	3.151
*P*	0.138	0.0001	0.280	0.001	0.002
Gestation time^b^					
Coefficient ± SE	0.022 ± 0.072	0.227 ± 0.072	0.034 ± 0.078	−0.039 ± 0.046	0.067 ± 0.120
*t*_226_	0.308	3.144	0.436	0.842	0.560
*P*	0.759	0.002	0.663	0.401	0.576

a The linear regression coefficients (i.e., P and C) describe the linear slope (given by ***β***_***i***_) of the relationship between nutrient intake and the response variable, whereas the quadratic regression coefficients (i.e., P × P and C × C) describes the curvature (given by ***γ***_***ii***_) of this relationship, with a negative ***γ***_***ii***_ indicating a convex relationship (i.e., a peak on the response surface) and a positive ***γ***_***ii***_ indicating a concave relationship (i.e., a trough on the response surface). The correlational regression coefficient (i.e., P × C) describes how the covariance between the two nutrients (***γ***_***ij***_) influences the response variable, with a negative ***γ***_***ij***_ indicating that a negative covariance between nutrients increases the response variable and a positive ***γ***_***ij***_ indicating that a positive covariance between nutrients increases the response variable. Full details of this approach are provided in Lande and Arnold (1983).

b The sign of standardized gestation time was reversed for analysis to make this response variable directly comparable to other traits (i.e., a short gestation time is good for reproductive potential).

**Table 2:** Sequential model building approach comparing the effects of P and C intake on traits within (A) male and (B) female *N*. *cinerea*. The sequential *F*‐tests test the differences in the sign and strength of the linear and nonlinear regression gradients across different response variables or the sexes. When significant differences in linear or quadratic regression gradients were detected, univariate tests were used to determine whether this overall effect was due to the daily intake of P and/or C. The angle (***θ***) between the linear vectors (and 95% CI) is also provided to highlight differences in the directionality of any observed nutrient effects

	SS_R_	SS_C_	DF_1_	DF_2_	*F*	*P*	***θ*** (95% CI)
(A) Males							
3H2B versus 2MT							
Linear	249.61	249.47	2	474	0.13	0.88	6.90° (0.00°,16.89°)
Quadratic	212.42	212.38	2	470	0.04	0.96	
Correlational	210.23	210.16	1	468	0.16	0.69	
3H2B versus 4E2M							
Linear	289.59	287.30	2	474	1.89	0.15	11.16° (0.00°,25.01°)
Quadratic	254.89	254.67	2	470	0.20	0.82	
Correlational	251.54	251.22	1	468	0.60	0.44	
2MT versus 4E2M							
Linear	287.64	285.41	2	474	1.85	0.16	9.73° (0.00°,22.61°)
Quadratic	252.98	252.81	2	470	0.16	0.85	
Correlational	248.69	248.00	1	468	1.30	0.25	
(B) Females							
Clutch size versus Gestation time							
Linear	419.51	418.00	2	448	0.81	0.45	19.29° (0.00°,51.85°)
Quadratic	412.11	411.13	2	444	0.53	0.59	
Correlational	408.35	402.36	1	442	6.58	0.011	

In females, clutch size increased linearly with daily C intake but was independent of P intake (Table 1). Clutch size peaked at around 8 mg of C per day, but there was not a significant peak in this trait with daily P intake (Fig. 1D; Table 1). There was also a significant negative correlational gradient indicating that clutch size is maximized on high C and low P diets (P:C = 1:8) (Fig. 1D; Table 1). Gestation time decreased linearly with daily intake of C but was not influenced by P intake (Fig. 1E; Table 1) or by quadratic or correlational effects of daily P and C (Table 1). Comparing nutritional landscapes for female reproductive traits showed that there were no significant differences in the linear or quadratic effects of daily P and C intake on clutch size and gestation time (Table 2). There was, however, a significant difference in the correlational effects of daily P and C intake on these traits. This was due to a significant negative correlational gradient for clutch size but not gestation time (Table 2). Despite this, the angle between the linear nutritional vectors for clutch size and gestation time was only 19.29°. This shows that clutch size is maximized and gestation time minimized in a similar region of the nutritional landscape (ca. 1:8 P:C) (Fig. 1D and E).

Comparing male and female reproductive traits, the sequential modeling approach showed significant differences in the linear effects of daily P and C intake on levels of male sex pheromones and female clutch size, but not in the quadratic or correlational effects of these nutrients (Table 3). For each sex pheromone, the difference in linear effects occurs because pheromone levels decrease with the daily P intake, whereas clutch size does not and also because pheromone levels are more responsive (i.e., steeper positive linear gradients) to the daily intake of C than clutch size (Table 3). The angles between the linear nutritional vectors for pheromone levels and clutch size ranged from 30.57° to 40.30°, which is low to moderate given that the maximal possible angle of separation is 180° (Table 3). This indicates that male pheromones and female clutch size occupy similar regions on the nutritional landscape (Fig. 1A–D).

**Table 3:** Sequential model building approach comparing the effects of P and C intake on traits across the sexes in *N*. *cinerea*. The angle (***θ***) between the linear vectors (and 95% CI) is also provided to highlight differences in the directionality of any observed nutrient effects

	SS_R_	SS_C_	DF_1_	DF_2_	*F*	*P*	***θ*** (95% CI)
3H2B versus Clutch size							
Linear	353.57	328.55	2	461	17.55	0.0001^A^	40.30° (16.68°,63.85°)
Quadratic	305.50	304.18	2	457	0.99	0.37	
Correlational	296.29	294.98	1	455	2.02	0.16	
2MT versus Clutch size							
Linear	351.55	326.66	2	461	17.56	0.0001^B^	38.13° (15.31°,61.29°)
Quadratic	303.78	302.32	2	457	1.10	0.33	
Correlational	293.10	292.36	1	455	1.15	0.28	
4E2M versus Clutch size							
Linear	376.82	364.38	2	461	7.87	0.0004^C^	30.57° (5.47°,53.60°)
Quadratic	345.42	344.61	2	457	0.54	0.58	
Correlational	333.69	333.42	1	455	0.37	0.54	
3H2B versus Gestation time							
Linear	369.10	340.92	2	461	19.05	0.0001^D^	30.36° (0.08°,57.37°)
Quadratic	325.51	321.19	2	457	3.07	0.047^E^	
Correlational	321.18	320.16	1	455	1.45	0.23	
2MT versus Gestation time							
Linear	367.55	339.03	2	461	19.39	0.0001^F^	28.65° (0.15°,54.67°)
Quadratic	323.65	319.33	2	457	3.09	0.046^G^	
Correlational	319.23	317.54	1	455	2.42	0.12	
4E2M versus Gestation time							
Linear	392.15	376.76	2	461	9.41	0.0001^H^	23.49° (0.00°,49.30°)
Quadratic	364.52	361.37	2	457	1.99	0.14	
Correlational	361.27	358.60	1	455	3.39	0.07	

Univariate tests: ^A^P: *F*_1,461_ = 15.66, *P *=* *0.0001, C: *F*_1,461_ = 4.56, *P *=* *0.033; ^B^P: *F*_1,461_ = 14.33, *P *=* *0.0002; C: *F*_1,461_ = 5.51, *P *=* *0.019; ^C^P: *F*_1,461_ = 6.49, *P *=* *0.011; C: *F*_1,461_ = 2.42, *P *=* *0.12; ^D^P: *F*_1,461_ = 8.79, *P *=* *0.0032; C: *F*_1,461_ = 12.11, *P *=* *0.0006; ^E^P: *F*_1,457_ = 0.16, *P *=* *0.69; C: *F*_1,457_ = 5.75, *P *=* *0.017; ^F^P: *F*_1,461_ = 7.80, *P *=* *0.005; C: *F*_1,461_ = 13.63, *P *=* *0.0002; ^G^P: *F*_1,457_ = 0.38, *P *=* *0.54; C: *F*_1,457_ = 5.46, *P *=* *0.020; ^H^P: *F*_1,461_ = 2.66, *P *=* *0.10; C: *F*_1,461_ = 8.10, *P *=* *0.0046.

A sequential modeling approach also showed significant differences in the linear effects of daily P and C intake on levels of male sex pheromones and female gestation time (Table 3). Again, this was because pheromone levels decrease with the daily P intake, whereas gestation time does not and because all three sex pheromones are more responsive to the daily intake of C than gestation time (Table 3). There was also a significant difference in the quadratic effects of daily P and C intake on the levels of two pheromones (3H2B and 2MT) and gestation time but not with the third pheromone (4E2M) (Table 3). In both cases, this difference was due to a peak in pheromone expression with daily C intake, but a peak did not exist for gestation time (Table 3). In contrast, there were no differences in the correlational effects of daily nutrient intake on pheromones and gestation time (Table 3). The angles between the linear nutritional vectors for pheromone levels and gestation time ranged from 23.49° to 30.36° (Table 3), which also indicates that pheromone levels and gestation time occupy similar regions of the nutritional landscape (Fig. 1A–C,E).

### Experiment 1: How do males and females regulate their nutrient intake when restricted to a single imbalanced diet?

**Figure 2:**
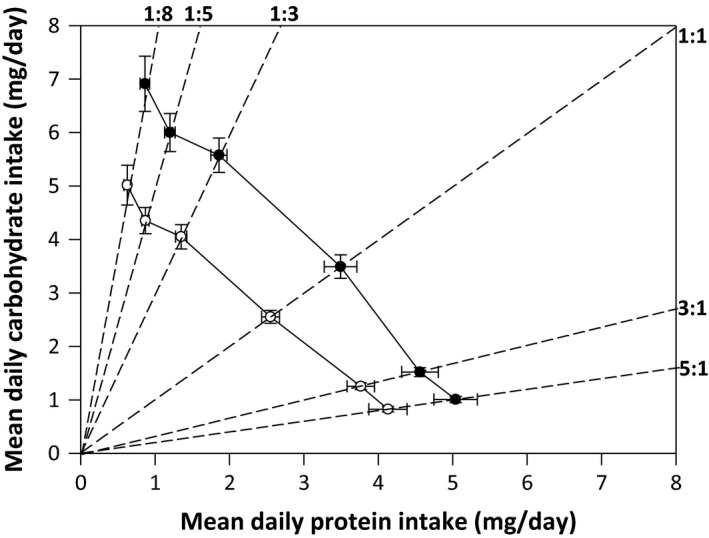
The mean (±SE) daily protein (P) and carbohydrate (C) intake for the male (open circles) and female (closed circles) cockroaches fed ad libitum amounts of diets along the six nutritional rails (dashed lines). The solid lines connecting the mean nutrient intake across rails for each sex create an intake array, the slope of which indicates the degree to which individuals prioritize their intake of nutrients when constrained to a single, nutritionally imbalanced diet.

An ANCOVA including sex as fixed effect, mean daily P intake as a covariate, and mean daily C intake as the response variable revealed a significant effect of sex on mean daily C intake (*F*_1,8_ = 79.982, *P *=* *0.0001) because females, on average, ingested more C per day than males (Fig. 2). There was an overall effect of mean daily P intake on the mean daily intake of C (*F*_1,8_ = 748.414, *P *=* *0.0001) with the slope of the intake array being significantly negative for both males and females (Fig. 2). Importantly, there was a significant interaction between sex and the mean daily intake of P (*F*_1,8_ = 5.471, *P *=* *0.047), indicating that the slope of the intake array differed significantly for the sexes. Regression analysis showed that the slope of the intake array was more negative for females than males (*β *± SE: females: −1.364 ± 0.075, *t*_5_ = 18.095, *r* ^2^ = 0.988, *P *=* *0.0001; males: −1.150 ± 0.043, *t*_5_ = 26.934, *r* ^2^ = 0.995, *P* = 0.0001) (Fig. 2). For both males (*t*_4_ = 3.488, *P *=* *0.025) and females (*t*_4_ = 4.867, *P *=* *0.008), the slope of the mean intake array was significantly greater than a hypothetical slope of −1, suggesting that the sexes are not regulating their intake based on the equality of nutrients or energy but are regulating their daily intake of P more strongly than their daily intake of C. In agreement with this, there was significantly greater variation in the daily intake of C than P in both males (*CV*_P_ = 0.741, *CV*_C_ = 0.687, *F*_239,239_ = 1.581, *P *=* *0.0002) and females (*CV*_P_ = 0.702, *CV*_C_ = 0.714, *F*_226,226_ = 2.161, *P *=* *0.0001). There was, however, no difference in the coefficient of variation (*CV*) for daily P (*F*_239,226_ = 1.056, *P *=* *0.340) or C (*F*_226,239_ = 1.039, *P *=* *0.384) intake between the sexes, suggesting that males and females regulate their intake of these nutrients in the same way.

### Experiment 2: How do males and females regulate their nutrient intake under dietary choice?

**Figure 3:**
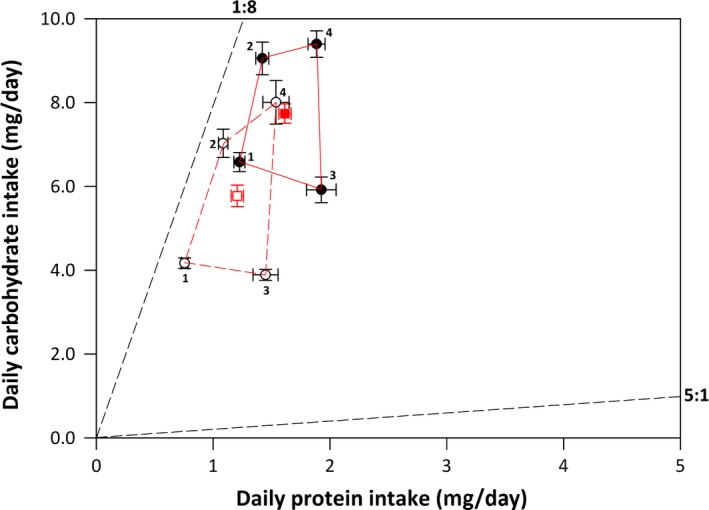
The mean (±SE) daily protein (P) and carbohydrate (C) intake for male (open symbols) and female (closed symbols) *N*. *cinerea* on each of the four diet pairs (indicated by numbers). The regulated intake point is also provided for males (open red square) and females (closed red square). The black dashed lines represent the nutritional rails that the alternate diets in each diet pair originate from and cockroaches can, in theory, regulate their intake of nutrients to any location within these rails. The total region in nutrient space that males (red, dashed) and females (red, solid) occupy through active dietary choice is also provided.

**Figure 4:**
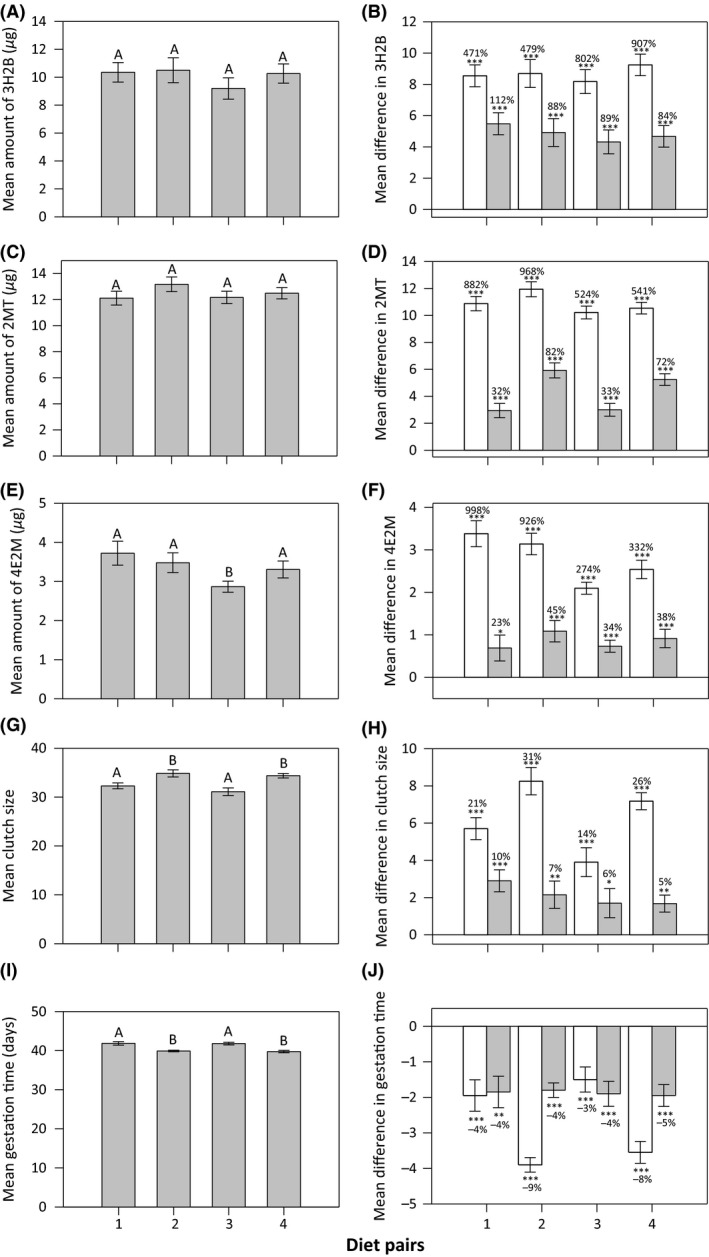
Mean (±SE) expression of the three male sex pheromones [(A) 3H2B, (C) 2MT, and (E) 4E2M], as well as female (G) clutch size and (I) gestation time in *N. cinerea* fed one the four alternate diet pairs. Diet pairs with different letters (above the bars) differ significantly in post hoc analysis. The mean (±SE) difference in (B) 3H2B, (D) 2MT, (F) 4E2M, (H) clutch size, and (J) gestation time when individuals are able to actively choose between diets and when they consume each diet in the pair exclusively (i.e., without choice), where the white bars represent the high protein diet in the pair (either diet 2 or 4, Table S1) and the gray bars represent the high carbohydrate diets in the pair (either diet 22 or 24, Table S1). Significance of these differences from a mean of zero (as determined by a one‐sample *t*‐test) is provided by asterisks, where *P <* *0.05, **P <* *0.01, and ***P <* *0.0001. Percentage increases or decreases from the mean when consuming only a single diet in the pair is added above each bar.

When given dietary choice, both males and females consumed more of the carbohydrate‐rich diet in each diet pair, irrespective of the total nutrient content of the diets contained in the pair (Fig. S2). This resulted in both sexes ingesting more C and less P than expected had they fed randomly (Fig. S2). MANOVA revealed that daily nutrient intake differed significantly across the sexes and diet pairs (Table 4). These overall multivariate effects were due to sex differences in the daily intake of both P and C, with females having a higher daily intake of both nutrients within each diet pair, as well as differences in the daily intake of both nutrients across diet pairs (Table 4, Fig. 3). The interaction between sex and diet pair, however, was not significant. This illustrates that the pattern of daily intake of nutrients on the different diet pairs was equivalent for the sexes (Table 4, Fig. 3). This is shown in Figure 4 where the daily intake of P and C on each of the diet pairs shows a similar pattern for both sexes. This pattern of nutrient results in a regulated intake point that is significantly higher in females than in males but both sexes follow the same nutritional rail (P:C ratio for males = 1:4.78; females = 1:4.79) (Fig. 3). We mapped the regulated intake point for each sex onto their nutritional landscapes to determine whether the sexes are able to maximize their reproductive traits through dietary regulation (Fig. 1). The regulated intake point for males was close to the P:C blend allowing optimal expression of all three sex pheromones (Fig. 1A–C). Likewise, the regulated intake point for females was close to the nutrient intake supporting optimal clutch size in females (Fig. 1D) but lower than the intake supporting the fastest gestation (Fig. 1E). There were no sex differences in the CV for daily C intake (*CV*_males_ = 0.397, *CV*_females_ = 0.396, *F*_79,79_ = 1.002, *P *=* *0.496) and daily P intake (*CV*_males_ = 0.394, *CV*_females_ = 0.291, *F*_79,79_ = 1.354, *P *=* *0.090) across diet pairs. This suggests that the sexes regulate their intake of P and C to the same extent under choice conditions.

**Table 4:** Multivariate analysis of variance (MANOVA) examining differences in the daily intake of P and C of male and female *N*. *cinerea* feeding on the four different diet pairs used in our choice experiment. To help interpret overall multivariate effects, we provide univariate ANOVAs for each term in the multivariate model

	MANOVA			
	Pillai's trace	*F*	df	*P* value
Sex (A)	0.348	40.304	2151	0.0001
Diet pair (B)	1.089	60.614	6304	0.0001
A × B	0.025	0.652	6304	0.691

	Nutrient	Univariate ANOVAs		
		*F*	df	*P* value
Sex (A)	P	49.715	1152	0.0001
	C	75.626	1152	0.0001
Diet pair (B)	P	36.725	3152	0.0001
	C	70.423	3152	0.0001
A × B	P	0.439	3152	0.725
	C	0.883	3152	0.451

### Experiment 2: The reproductive consequences of nutrient regulation under dietary choice

MANOVA revealed that there was no overall multivariate effect of diet pair on the expression of male sex pheromones (Pillai's trace = 0.150, *F*_9,228_ = 1.330, *P *=* *0.222). This finding was confirmed by univariate ANOVAs on each of the sex pheromones (3H2B: *F*_3,76_ = 0.610, *P *=* *0.611; 2MT: *F*_3,76_ = 0.950, *P *=* *0.421; 4E2M: *F*_3,76_ = 2.328, *P *=* *0.081), although post hoc analysis did show that levels of 4E2M were significantly lower on diet pair 3 than the other diet pairs (Fig. 4A,C and E). This lack of difference in pheromone expression across diet pairs is not surprising given that regulated intake points fell near the optima for pheromone expression on the nutritional landscape (Fig. 1A–C). In contrast, MANOVA showed a significant multivariate effect of diet pair on reproductive traits in females (Pillai's trace = 0.473, *F*_6,144_ = 7.432, *P *=* *0.0001) and univariate ANOVAs confirmed this was due to significant differences in both clutch size (*F*_3,72_ = 7.012, *P *=* *0.0001) and gestation time (*F*_3,72_ = 11.269, *P *=* *0.0001) across diet pairs. Post hoc analysis showed that clutch size was significantly larger and gestation time significantly shorter on diet pairs 2 and 4 compared to diet pairs 1 and 3 (Fig. 4G and I). This pattern can be explained by the fact that the mean nutrient intake on diet pairs 2 and 4 is closer to the optima for clutch size and the shortest gestation time on the nutritional landscapes than diet pairs 1 and 3 (Fig. 1D and E).

To examine how dietary choice affects reproductive traits in the sexes, we compared the trait expression when cockroaches were allowed to actively choose between diets in a given pair to the mean trait expression in cockroaches that were exclusively fed on either diet in the pair. For males, the expression of 3H2B (Fig. 4B), 2MT (Fig. 4D), and 4E2M (Fig. 4F) were all greater with dietary choice, and this increase was consistently greater when compared to the exclusive consumption of the higher P diet in the diet pair. Across diet pairs, the average increase in pheromone expression when compared to the high P and C diets in the pair was 665% and 93% for 3H2B, 729% and 55% for 2MT, and 633% and 35% for 4E2M, respectively (Fig. 4B,D and F). In females, clutch sizes were larger (Fig. 4H) and gestation time shorter (Fig. 4J) with active dietary choice. As for males, this change in trait expression was greatest when compared to the exclusive consumption of the higher P diet in the diet pair. Across diet pairs, the average increase in clutch size when compared to the high P and C diets in the pair was 23% and 7%, whereas the average decrease in gestation time was 6% and 4%, respectively (Fig. 4H and J). Dietary choice also significantly influenced the variation observed for reproductive traits in males and females. The *CV* for all three male sex pheromones, as well as clutch size and gestation time in females, was significantly lower when cockroaches were given dietary choice compared to when cockroaches were fed exclusively on either diet in the pair (Table S2). In males, the average reduction in the *CV* for pheromone expression when compared to the high P and C diets in the pair was 68% and 69% for 3H2B, 70% and 72% for 2MT, and 67% and 68% for 4E2M, respectively (Table S2). In females, the average reduction in the *CV* when compared to the high P and C diets in the pair was 74% and 70% for clutch size and 70% and 68% for gestation time, respectively (Table S2). Collectively, these analyses clearly demonstrate that active dietary choice increases the mean and reduces the variance in important reproductive traits relative to feeding exclusively on a single, imbalanced diet.

Males produced significantly higher levels of 3H2B when given dietary choice compared to the single best diet within the landscape (*t*_9_ = 4.391, *P *=* *0.002), but levels of 2MT and 4E2M did not differ significantly (2MT: *t*_9_ = 1.412, *P *=* *0.192; 4E2M: *t*_9_ = 0.923, *P *=* *0.380) (Fig. S3). In contrast, females produced significantly larger clutch sizes when restricted to the single best diet compared to dietary choice (*t*_9_ = 5.044, *P *=* *0.001), but gestation time did not differ significantly (*t*_9_ = 1.215, *P *=* *0.255) (Fig. S3). In total, the percentage of males in our no‐choice experiment that had pheromone levels exceeding the mean across diet choice pairs was much lower (3H2B = 0%, 2MT = 4%, 4E2M = 9%) than observed for female reproductive traits (clutch size = 21%, gestation time = 31%).

## Discussion

An individual's reproductive success often depends on the amount and ratio of nutrients that it consumes (Simpson and Raubenheimer 2012). This is because many reproductive traits, including female fecundity (Lee et al. 2008; Harrison et al. 2014; Jensen et al. 2015a) and male sperm production (Bunning et al. 2015), sexual advertisement (Maklakov et al. 2008; Harrison et al. 2014) and sexual trait expression (South et al. 2011; Sentinella et al. 2013; House et al. 2015), are often maximized in individuals that eat specific combinations of nutrients. In the few species where the effects of nutrients on reproduction have been examined in both sexes (e.g. Maklakov et al. 2008; Harrison et al. 2014; Jensen et al. 2015a), the intake of nutrients that is optimal for reproduction in males and females differs. However, more studies on the sex‐specific effects of nutrients on reproduction are needed before any general conclusions can be made. Furthermore, it is unclear how the sexes regulate their intake of nutrients and the effect that nutrient balancing under dietary choice has on their subsequent fitness. In this study, we constrained male and female cockroaches (*Nauphoeta cinerea*) to single diets varying in P and C content to assess how intake of these nutrients influences key reproductive traits and whether the sexes differentially prioritize intake of these nutrients. Next, we determined how the sexes regulate their intake of nutrients under dietary choice and the effect that this regulation has on their reproductive performance. In contrast to the insects studied to date where the sexes have different nutritional optima for reproduction, male and female *N. cinerea* maximized reproductive traits when consuming large amounts of P:C in a 1:8 ratio. Furthermore, both sexes prioritized regulating the daily intake of P over C when constrained to a single diet, although this prioritization was slightly stronger in females than males. Under dietary choice, both sexes actively regulated their intake of nutrients at a P:C ratio of 1:4.8. Although this ratio is not perfectly aligned with the dietary optima for any of the reproductive traits we measured on single diets, male and female cockroaches that regulated their nutrition increased the mean and reduced the variance in reproductive performance. This effect was especially large for male pheromone expression. This provides clear support for the balanced diet hypothesis and demonstrates that nutrient balancing, rather than any single nutrient blend, is key to maximizing reproductive success in this species.

We found that the expression of all three male pheromones, and therefore male attractiveness (Clark et al. 1997; Moore and Moore 1999; South et al. 2011), increased with the daily intake of C but declined with the daily intake of P. Consequently, the expression of all three pheromones was maximized at a high intake of nutrients in a P:C ratio of 1:8. This finding is remarkably similar to our earlier study on male *N. cinerea* using the same geometric array of diets (South et al. 2011), where pheromone expression and male attractiveness were also maximized around high intake of the 1:8 P:C diet. Given that lipids appear to be the major precursor for the biosynthesis of sex pheromone in cockroaches (Chase et al. 1990, 1992; Schal et al. 1991) and that lipid storage increases with C intake in male *N*. *cinerea* (South et al. 2011), the positive relationship between C intake and pheromone expression we document may represent a direct mechanistic link.

Despite the similarities between these studies, however, there are a number of subtle differences that are likely to reflect the fact that pheromones were sampled from much older males in our current study (55 days posteclosion) compared to South et al. (2011) (10 days posteclosion). First, South et al. (2011) showed that P intake had little effect on pheromone expression, whereas we found that pheromone expression significantly decreased with intake of this nutrient. This suggests that a much higher cumulative intake of P is required before the deleterious effects of this nutrient on pheromone expression are realized. While it is unclear exactly how P intake decreases pheromone expression in *N. cinerea*, it is possible that P‐induced satiety reduces lipid storage and therefore the subsequent capacity to produce pheromones. While the effects of P‐induced satiety on lipid storage are well documented in rodents (Lacroix et al. 2004; Sørensen et al. 2008) and humans (Weigle et al. 2005), comparably little is known about this in insects. Consistent with this idea, male *N*. *cinerea* eat far less when restricted to high P diets (Fig. 1A–C) and both lipid storage and pheromone expression are reduced in high P regions of the nutritional landscape (South et al. 2011). Second, South et al. (2011) documented differences in the linear and nonlinear effects of C intake on pheromone expression. For example, 3H2B expression was more responsive to the intake of C than 2MT and 4E2M and there was no peak in 2MT expression with C intake (South et al. 2011). In contrast, we found little difference in the linear or nonlinear effects of nutrient intake on pheromone expression, as evidenced by lack of difference in our sequential models and the small angles between the linear nutritional vectors (5.75–8.54°). This suggests that the effects of C intake on pheromones become more similar with age in male *N. cinerea*. Finally, both mean pheromone levels and the slopes of the standardized nutritional gradients for C intake are greater in our current study than in South et al. (2011). This suggests that mean pheromone levels not only increase with male age, but males also become more responsive to the intake of this nutrient over time. This contradicts earlier work on this species which shows that pheromone levels peak around 15–20 days posteclosion and decline thereafter, with pheromone levels at 50 days posteclosion being similar to levels at 10 days posteclosion (i.e., sexual maturity) (Moore et al. 1995). It is important to note, however, that diet was not manipulated in this experiment and all males received a standard laboratory diet, which likely contain relatively high amounts of P.

As for male reproductive traits, gestation time and clutch size in females were also highly dependent on nutrient intake. Gestation time was shortest and clutch size largest when consuming a high intake of nutrients in a P:C ratio of 1:8. Consequently, females are able to maximize the expression of both reproductive traits when consuming a high C, low P diet. Although both P and C contain the same energy content per gram ingested (~16.7 kJ/g), C is an easier source of energy to utilize than P (Cohen 2015). The reliance of clutch size and gestation time on high C intake suggests that these reproductive traits are energetically costly to express. Supporting this argument, gestation time is negatively correlated with standard metabolic rate in female *N. cinerea* (Schimpf et al. 2012). Surprisingly, Schimpf et al. (2012) also found that metabolic rate was unrelated to clutch size, although this result should be interpreted with some caution as metabolic rate was not compared to virgin females and natural variation in clutch size for the reproducing females examined was not reported (Schimpf et al. 2012).

Formal comparison of the nutritional landscapes revealed subtle differences in the effects of P and C on reproductive traits in males and females. Most notable was the fact that all three male pheromones were more responsive (i.e., steeper nutritional gradients) to the intake of C than female reproductive traits and also that P intake had a negative effect on male pheromone expression but not on female reproductive traits. However, the small angles (28.65° to 40.30°) between nutritional vectors for male pheromone expression, female clutch size, and gestation time demonstrate that all traits peaked in similar regions of the nutritional landscape and therefore that both sexes require a similar intake of nutrients to maximize the expression of these sex‐specific reproductive traits. This result is surprising and differs markedly from most other studies on insects using the GF (Maklakov et al. 2008; Harrison et al. 2014; Jensen et al. 2015a) that show that males and females need to eat different diets to maximize their reproductive effort. Typically, females must consume more P than males to maximize reproduction. For example, in field crickets egg laying is greatest in females that consume large amounts of diet with a P:C ratio of 1:1 (*T. commodus*; Maklakov et al. 2008) or 3:1 (*G. veletis*; Harrison et al. 2014). In contrast, male *T. commodus* maximizes sexual advertisement when consuming a high intake of diet in a P:C ratio of approximately 1:8 (Maklakov et al. 2008) and male *G. veletis* when consuming high‐energy diets (Harrison et al. 2014). Likewise, female *D. melanogaster* maximize egg laying rates when they eat a 1:2 P:C ratio (Lee et al. 2008; Jensen et al. 2015a), while the number of offspring sired by a male is greatest when consuming a P:C ratio of 1:16 (Jensen et al. 2015a). Our results therefore show that relative to females from other insect species, female *N. cinerea* have a low P requirement for reproduction.

There are a number of possible explanations for why female *N. cinerea* have such a low P requirement for reproduction. First, this may represent an adaptation to maintain nutritional homeostasis in cockroaches. Most cockroach species, including *N. cinerea* (Kambhampati et al. 2013)*,* harbor endosymbiotic bacteria (*Blattabacterium*) within specialized cells in the fat body that enable them to store nitrogen as uric acid during times of excess P and recycle the nitrogen for amino acids when P is limited in the diet (Sabree et al. 2009; Patiño‐Navarrete et al. 2014). In our experiment, cockroaches were fed commercial rat chow, which contains relatively large amounts of crude P (~20%), during their juvenile development. The low P requirements we observe in adult females may reflect that females stored excess nitrogen as a juvenile and, with the help of endosymbionts, recycled it as adults when consuming diets with low P content. This hypothesis is clearly speculative and requires testing empirically, but hints at the role endosymbionts may play in altering the relationship between nutrient intake and reproductive performance. A second explanation for the low P requirement of females is that we only measured the nutrient requirements of females during the production of her first clutch of offspring. Female *N. cinerea* produce multiple clutches in their lifetime and are long‐lived, living up to 3 years in captivity (Moore and Moore 2001). It is possible that females possess enough P to produce a single clutch but that P requirements increase as females produce more offspring and deplete their nitrogen reserves. In support of this idea, an age‐dependent increase in P intake occurs in honeybees (*Apis mellifera scutellata*) when workers begin nursing larvae (Archer et al. 2014). Clearly, further empirical studies are needed to test the possible effect of nutrient storage and recycling on nutrient balancing in *N. cinerea* and other cockroaches.

Given that the reproductive traits measured in our study have similar dietary optima in the sexes, it is not surprising that males and females regulated their nutrition in a similar way. When restricted to a nutritionally imbalanced diet, individuals must find a suitable compromise between overingesting some nutrients and underingesting others: This is known as the “rule of best compromise” (Simpson and Raubenheimer 1997). The outcome of this regulation should reflect the relative costs and benefits of overingesting one of the nutrients and underingesting the other (Simpson and Raubenheimer 1997). When we restricted cockroaches to a single imbalanced diet, both sexes exhibited negative intake arrays with slopes significantly steeper than −1, indicating that they regulated their intake of P more tightly than their intake of C. Inspection of the nutritional landscapes provides justification for this regulation as the overingestion of P reduces reproductive performance in both sexes; by tightly regulating their intake of P, males and females may be able to avoid these costs. The prioritization of regulating P over C intake on imbalanced diets appears commonplace, being demonstrated in species including cockroaches (Jensen et al. 2015b), cabbage loopers (*Trichoplusia ni*, Shikano and Cory 2014), and mice (Sørensen et al. 2008). Few studies have, however, examined sex differences in the prioritization of these nutrients. Consistent with our finding, male and female German cockroach (*B. germanica*) nymphs show similar prioritization for the intake of P over C (Raubenheimer and Jones 2006). This contrasts recent work, however, on mealworms (*Tenebrio molitor*) where both sexes pioritize the intake of C over P (Rho and Lee 2014) and the field cricket *G. veletis* where the sexes give equal priority to P and C intake (Harrison et al. 2014). We also found that the sexes showed a similar strategy of nutrient regulation when given the choice between alternate diet pairs. Both males and females consumed diets in a nonrandom manner and regulated their intake point at a P:C ratio of 1:4.8. This regulated intake point is similar to those observed in our previous studies on male *N. cinerea*. Using the same pairs of diets as our current study, Bunning et al. (2015) found that males regulate toward an almost identical intake of nutrients (P:C = 1:4.95). South et al. (2011) tested a narrower range of diets in their dietary choice experiment (P:C rails of 1:8 and 1:1) compared to our current study (1:8 and 5:1) but also found that males regulated toward a greater relative intake of C (P:C = 1:3.2). The regulated intake point we document in *N. cinerea* is more C biased than that seen in another cockroach species (female *B. germanica*, ca. P:C = 1:2, Jensen et al. 2015b) but is similar to the intake targets reported in crickets (e.g., male and female *T. commodu*s, P:C = 1:3, Maklakov et al. 2008; male *G. veletis*, P:C = 1:4.1, Harrison et al. 2014).

The regulated intake point we observed was more P biased than optimal for reproductive traits, suggesting that *N. cinerea* is not feeding to optimize the expression of these traits. This is despite cockroaches being offered a high carbohydrate diet in their choice pairs that closely aligned with their dietary optima. There are several possible reasons for this. In *N. cinerea*, males produce more sperm and sire more offspring when fed a P:C ratio of 1:2 (Bunning et al. 2015). Males may regulate their intake of P and C to eat a diet that allows moderate pheromone expression and sperm production (Bunning et al. 2015). If dietary choice is regulated by the same gene(s) in both sexes, then this selection on male intake toward a higher relative intake of P could, in theory, also drag female intake toward a high protein food, even if this has costs to female fitness: a process known as intralocus sexual conflict (IASC) over nutrient intake. IASC occurs whenever the same gene(s) control shared trait(s) in the sexes, but these trait(s) are subject to contrasting patterns of selection in the sexes. This can lead to an evolutionary tug‐of‐war, which may prevent one or both sexes from evolving to their nutritional optimum (Bonduriansky and Chenoweth 2009). It has been proposed that IASC over nutrient intake explains why male and female Australian field crickets (*T. commodus*, Maklakov et al. 2008) and *D. melanogaster* (Jensen et al. 2015a) share a regulated intake point but have very different dietary optima for reproductive effort. However, in the only formal test of this process, Reddiex et al. (2013) found little support for IASC in *D. melanogaster* because the contrasting effects of P and C intake on reproduction in the sexes were well aligned with the major axis of genetic (co)variance within and between the sexes for the intake of these nutrients. Alternatively, the higher than expected regulated intake of P may reflect the fact that the sexes are attempting to maximize the expression of a shared trait that was not measured in our study. One possible candidate is immune function, which is enhanced with P intake in many species (e.g., Povey et al. 2009; Cotter et al. 2011). Given that immune function is likely to be an important determinant of fitness in natural populations (Rolff and Reynolds 2009), it is possible that both sexes regulate their intake of nutrients to balance the trade‐off between immune function and reproduction. More work is needed, however, to test this and also whether IASC contributes to the poor alignment between the regulated intake point and the optima for reproductive traits.

Despite the poor alignment of the regulated intake point with the peaks of the nutritional landscapes for reproductive traits, when given a choice between alternate diets cockroaches increased mean trait expression relative to individuals constrained to either diet in the pair and reduced variance in trait expression. Our findings are therefore in general agreement with the balanced diet hypothesis (DeMott 1998). It is well known that the dietary requirements of individuals in a population can vary with a variety of factors, including age (Paoli et al. 2014), sex (e.g., Maklakov et al. 2008; Harrison et al. 2014; Jensen et al. 2015a), pathogen infection (e.g., Povey et al. 2009), exposure to toxins (e.g., Archer et al. 2014), predators (e.g., Hawlena and Schmitz 2010), and past nutrient deficiencies (e.g., Jensen et al. 2014). Nutrient balancing may enable individuals to meet their specific dietary requirements, facilitating an increase in the mean of fitness‐related traits in the population and a reduction in the variance of those traits (Lefcheck et al. 2013; Lihoreau et al. 2015; Senior et al. 2015; Simpson et al. 2015). Recent meta‐analyses comparing the mean of fitness‐related traits (i.e., size, reproduction, life span) of generalist consumers when constrained to a single diet or when given a choice of diets support this argument and suggest that the positive effects of nutrient balancing may be a general feature of ecological systems (Lefcheck et al. 2013; Senior et al. 2015). Both studies found that, on average, consumption of a mixed diet increased the mean of fitness‐related traits. Senior et al. (2015) also found that a mixed diet reduced the variance in fitness‐related traits, especially as the availability of multiple diets increased.

While our findings are largely consistent with these meta‐analyses, there are some subtle differences. First, Lefcheck et al. (2013) found that although dietary choice, on average, increased mean fitness, they also found that the fitness of individuals consuming a mixed diet was not significantly greater than individuals only consuming the single best diet. Our results show that this is true for some reproductive traits in *N. cinerea*. Mean levels of 2MT and 4E2M in males and gestation time in females did not differ significantly between individuals consuming a mixed diet or when restricted to the single best diet, supporting the findings of Lefcheck et al. (2013). In contrast, mean clutch size was significantly greater in females consuming the single best diet compared to those given a mixed diet, whereas the reverse pattern was true for mean levels of 3H2B. Despite this, the number of individuals in our no‐choice experiment that produced reproductive traits exceeding the mean taken across our choice diet pairs was generally low, although much lower in males (3H2B = 0%, 2MT = 4% and 4E2M = 9%) than females (clutch size = 21%, gestation time = 31%). Consequently, while our results suggest that the consumption of a mixed diet is, on average, beneficial to the expression of reproductive traits in *N. cinerea*, our work also raises caution over which diet should be used for comparison when examining the benefits of consuming a mixed diet. When compared to the single best diet, consumption of a mixed diet had a much smaller effect on reproductive trait expression in the sexes than the broader range of diets used in our choice experiment. Second, Lefcheck et al. (2013) and Senior et al. (2015) found that the consumption of a mixed diet increased reproductive traits by 65% and 32%, respectively, over the consumption of a single diet. The increase in mean clutch size (higher P diet = 23%, higher C diet = 7%) and the reduction in mean gestation time (higher P diet = 6%, higher C diet = 4%) we observe in females consuming a mixed diet is smaller than these reported values. In contrast, while the increase in mean pheromone expression on a mixed diet relative to the higher P diet in the pair was within the range of values reported in Lefcheck et al. (2013) and Senior et al. (2015) (3H2B = 93%, 2MT = 55%, 4E2M = 35%), the observed increase relative to the higher C diet in the pair was markedly greater (3H2B = 665%, 2MT = 729%, 4E2M = 633%). Thus, our findings show that male pheromone expression in *N. cinerea* is extremely responsive to the intake of a mixed diet, which is consistent with earlier work on this species showing this reproductive trait is highly sensitive to the nutritional (Clark et al. 1997; South et al. 2011) and social (Moore et al. 1997; Moore and Moore 1999) environment. Finally, Senior et al. (2015) also found that the consumption of a mixed diet decreased the variation in reproductive traits by an average of 37% relative to the consumption of a single diet. The reduction we show in the *CV*s for reproductive traits was much higher than this value and was surprisingly consistent across diets, trait types, and the sexes. In males, the average reduction in the *CV* for pheromone expression when consuming a mixed diet compared to the higher P diet in the choice pair was 68%, 70%, and 67% for 3H2B, 2MT, and 4E2M, respectively, and 69%, 72%, and 68%, respectively, when compared to the higher C diet in the choice pair. For females, the average reduction in *CV* was 74% and 70% for clutch size and gestation time when compared to the higher P diet in the choice pair and 70% and 68% when compared to the higher C diet in the choice pair. This suggests that the observed reduction in the variation of reproductive traits when consuming a mixed diet may be more central to fitness in male and female *N. cinerea* than the increase in the mean of these traits, a view that is supported by at least some theoretical models (Gillespie 1974; Smith and Fretwell 1974; Sargent et al. 1987).

In conclusion, our results show that male and female *N. cinerea* are able to maximize reproductive traits when consuming low P, high C diets in a P:C ratio of approximately 1:8. This similarity in nutritional requirements across the sexes contrasts previous nutritional studies on invertebrates where females typically require a higher intake of P to maximize reproduction (Maklakov et al. 2008; Jensen et al. 2015a) and may represent the unique ability of cockroaches to recycle nitrogen from uric acid using endosymbiotic bacteria. Given these similar nutritional requirements, the sexes also regulated their intake of nutrients in a similar manner. When restricted to a single diet, both sexes prioritized regulating the intake of P over C and when given dietary choice, shared a common regulated intake point at a P:C ratio of 1:4.8. This regulated intake point was poorly aligned with the peaks on the nutritional landscapes indicating that the sexes are not optimally regulating their intake of nutrients to maximize reproductive traits. Dietary choice and a mixed intake of nutrients was shown to increase the mean and reduce the variation in all reproductive traits measured in the sexes compared to feeding exclusively on either diet in the choice pair. This pattern was less clear, however, when comparing the reproductive traits of individuals consuming a mixed diet to those restricted to the consumption of the single best diet. Collectively, our results show that the intake of nutrients is key to male and female reproduction in *N. cinerea* and that the sexes regulate their intake of these nutrients in a similar way with and without dietary choice. Furthermore, our work also suggests that a mixed intake of nutrients is likely to be key to reproduction in *N*. *cinerea,* but this relationship is likely to be complex in nature.

## Conflict of Interest

None declared.

## Supporting information


**Table S1.** Protein and carbohydrate composition of the 24 artificial diets used in our “no‐choice” feeding experiments.
**Figure S1.** The location of the 24 artificial diets used in our feeding experiments in nutritional space.
**Text S1**. Multivariate response surface approach used to characterize the nutritional landscapes for our three response variables.
**Text S2.** Sequential model building approach to compare nutritional landscapes across reproductive traits.
**Text S3.** Calculating the angle between linear nutritional vectors and their 95% CI.
**Figure S2.** The mean (±SE) daily amount of diet consumed and the deviance in the intake of protein and carbohydrates in each diet pair from random feeding in the sexes.
**Table S2.** *F* ratio tests comparing the Coefficient of Variation (*CV*) for reproductive traits in the sexes when given the opportunity to choose between alternate diets versus when feeding exclusively on a single diet.
**Figure S3.** The mean (±SE) reproductive traits in male and female *N. cinerea* across the 24 different artificial diets used in our no‐choice experiment.
