# Supplementary material for: Dietary choice for a balanced nutrient intake increases the mean and reduces the variance in the reproductive performance of male and female cockroaches
Source: Ecol Evol. 2016 Jun 12;6(14):4711–30. doi: 10.1002/ece3.2243 (PMC4979701; doi:10.1002/ece3.2243)
Supplement: Supplementary file 1 — Table S1. Protein and carbohydrate composition of the 24 artificial diets used in our “no‐choice” feeding experiments. Figure S1. The location of the 24 artificial diets used in our feeding experiments in nutritional space. Text S1. Multivariate response surface approach used to characterize the nutritional landscapes for our three response variables. Text S2. Sequential model building approach to compare nutritional landscapes across reproductive traits. Text S3. Calculating the angle between linear nutritional vectors and their 95% CI. Figure S2. The mean (±SE) daily amount of diet consumed and the deviance in the intake of protein and carbohydrates in each diet pair from random feeding in the sexes. Table S2. F ratio tests comparing the Coefficient of Variation (CV) for reproductive traits in the sexes when given the opportunity to choose between alternate diets versus when feeding exclusively on a single diet. Figure S3. The mean (±SE) reproductive traits in male and female N. cinerea across the 24 different artificial diets used in our no‐choice experiment. [file ECE3-6-4711-s001.docx]

**Supporting Information**

**Dietary choice for a balanced nutrient intake increases the mean and reduces the variance in the reproductive performance of male and female cockroaches**

Harriet Bunning^1^, Lee Bassett^1^, Christina Clowser^1^, James Rapkin^1^, Kim Jensen^1,2^, Clarissa M. House^1^, C. Ruth Archer^1,3^, John Hunt^1^

**Table S1.** Protein and carbohydrate composition of the 24 artificial diets used in our “no-choice” feeding experiments.

**Figure S1.** The location of the 24 artificial diets used in our feeding experiments in nutritional space.

**Text S1**. Multivariate response surface approach used to characterize the nutritional landscapes for our three response variables.

**Text S2.** Sequential model building approach to compare nutritional landscapes across reproductive traits.

**Text S3.** Calculating the angle between linear nutritional vectors and their 95% CI.

**Figure S2.** The mean (±SE) daily amount of diet consumed and the deviance in the intake of protein and carbohydrates in each diet pair from random feeding in the sexes.

**Table S2.** *F* ratio tests comparing the Coefficient of Variation (*CV*) for reproductive traits in the sexes when given the opportunity to choose between alternate diets versus when feeding exclusively on a single diet.

**Figure S3.** The mean (±SE) reproductive traits in male and female *N. cinerea* across the 24 different artificial diets used in our no-choice experiment.

**Table S1.** Protein (P) and carbohydrate (C) composition of the 24 artificial diets used in our “no-choice” feeding experiments. The total nutrients present in each diet are given as the sum of the percentage P and percentage C, with the remaining percentage consisting of indigestible crystalline cellulose.

| **Protein (P)** | **Carbohydrate (C)** | **P + C (%)** | **P : C** | **Diet Number** |
| --- | --- | --- | --- | --- |
| 10 | 2 | 12 | 5:1 | 1 |
| 30 | 6 | 36 | 5:1 | 2 |
| 50 | 10 | 60 | 5:1 | 3 |
| 70 | 14 | 84 | 5:1 | 4 |
| 9 | 3 | 12 | 3:1 | 5 |
| 27 | 9 | 36 | 3:1 | 6 |
| 45 | 15 | 60 | 3:1 | 7 |
| 63 | 21 | 84 | 3:1 | 8 |
| 6 | 6 | 12 | 1:1 | 9 |
| 18 | 18 | 36 | 1:1 | 10 |
| 30 | 30 | 60 | 1:1 | 11 |
| 42 | 42 | 84 | 1:1 | 12 |
| 3 | 9 | 12 | 1:3 | 13 |
| 9 | 27 | 36 | 1:3 | 14 |
| 15 | 45 | 60 | 1:3 | 15 |
| 21 | 63 | 84 | 1:3 | 16 |
| 2 | 10 | 12 | 1:5 | 17 |
| 6 | 30 | 36 | 1:5 | 18 |
| 10 | 50 | 60 | 1:5 | 19 |
| 14 | 70 | 84 | 1:5 | 20 |
| 1.33 | 10.66 | 12 | 1:8 | 21 |
| 4 | 32 | 36 | 1:8 | 22 |
| 6.66 | 53.55 | 60 | 1:8 | 23 |
| 9.33 | 74.66 | 84 | 1:8 | 24 |

**Figure S1.** The location of the 24 artificial diets used in our feeding experiments in nutritional space. Filled circles correspond to diets used in the no-choice experiment (Experiment 1). The open squares represent diets that were used in both the no-choice experiment (Experiment 1) and the choice trial (Experiment 2). Dotted lines show nutritional rails, which represent the ratio of protein to carbohydrate in each diet. These rails are labeled with their P:C content.


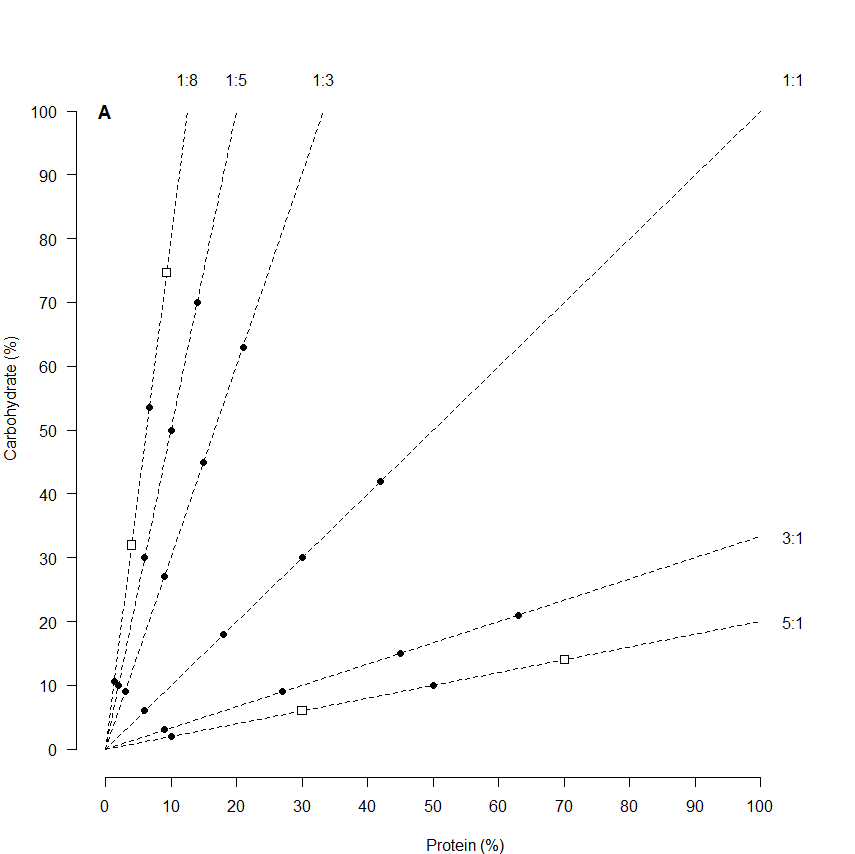


**Text S1**. **Multivariate response surface approach used to characterize the nutritional landscapes for our three response variables.**

We used a multivariate response surface approach to examine the effects of protein (P) and carbohydrates (C) intake on our reproductive traits (i.e. pheromone levels in males, clutch size and gestation time in females). This approach is based on the methodologies of Lande & Arnold (1983). Prior to analysis, we standardized each response variable and nutrient intake to a mean of zero and standard deviation of one using a *Z*- transformation to ensure that our regression gradients were presented in the same scale. We also reversed the sign of standardized gestation time, as female with faster gestation have a higher reproductive potential (see manuscript for more details). First, the following linear multiple regression model was fitted to estimate the linear gradients for P and C intake on each response variable:

**$R=\alpha+\beta P+\beta C+\varepsilon$** (Eq.1)

where *R* is the response variable, **a** is the regression intercept, ***β***s represent the partial regression gradients and **ɛ** is the random error component.

To estimate the nonlinear (i.e. quadratic and correlational) gradients for nutrient intake on the response variables, the following nonlinear multiple regression model was fitted:

**$R=\alpha+\beta P+\beta C+\gamma P^{2}+\gamma C^{2}+\gamma PC+\varepsilon$** (Eq.2)

where **γ***P^2^* and **γ***C^2^* represent the quadratic gradients for P and C, respectively and **γ***PC* represents the correlational gradient for these two macronutrients. For the quadratic gradients, a negative term indicates a peak on the nutritional landscape, whereas a positive term indicates a trough. The linear terms are included but not interpreted from Eq. 2: they are simply included so that the nonlinear effects can be examined when the linear effects have been removed.

**References**

Lande R, Arnold SJ (1983) The measurement of selection on correlated characters. *Evolution* 37: 1210-1226.

**Text S2.** **Sequential model building approach to compare nutritional landscapes across reproductive traits.**

We used a sequential model building approach to assess whether the linear and nonlinear effects of protein and carbohydrate ingestion differed for our response variables (Draper and John 1988; Chenoweth and Blows 2005). As our different responses variables (sperm number and offspring number) were measured in different scales, it was necessary to standardize them for statistical comparison. Prior to comparison, we therefore standardized each response variable and nutrient intake to a mean of zero and standard deviation of one using a *Z*- transformation. We then included a dummy variable, response type (*RT*), in a reduced model containing only the standardized linear terms:

 (Eq.1)

where *R* is our standardized response variables, *N_i_* refers to the intake of the *i*th nutrient, *n* represents the number of nutrients contained in the model and *ε* is the unexplained error. From (1), the unexplained (i.e. residual) sums of squares for this reduced model (*SS_r_*) was compared to the same quantity (*SS_c_*) from a second (complete) model that included all of the terms in (1) with the addition of the terms *α_i_N_i_RT* which represents the linear interaction of *RT* and the *i*th nutrient.

**$R=\beta_{0}+\alpha_{0}RT+\sum_{i=1}^{n} \beta_{i}N_{i}+\sum_{i=1}^{n} \alpha_{i}N_{i}RT+\varepsilon$** (Eq.2)

A partial *F*-test (Bowerman and O'Connell 1990) was used to compare *SS_r_* and *SS_c_* from (Eq.1) and (Eq.2) respectively:

**$F_{a,b}=\frac{(SS_{r}-SS_{c})/a}{SS_{c}/b}$** (Eq.3)

where *a* is the number of terms that differ between the reduced and complete model and *b* is the error degrees of freedom for *SS_c_*.

To test whether the quadratic effect of nutrient intake differed between response variables, the *SS_r_* from the reduced model:

$R=\beta_{0}+\alpha_{0}RT+\sum_{i=1}^{n} \beta_{i}N_{i}+\sum_{i=1}^{n} \alpha_{i}N_{i}RT+\sum_{i=1}^{n} \beta_{i}N_{i}^{2}+\varepsilon$ (Eq.4)

was compared to the *SS_c_* of the complete model:

$R=\beta_{0}+\alpha_{0}RT+\sum_{i=1}^{n} \beta_{i}N_{i}+\sum_{i=1}^{n} \alpha_{i}N_{i}RT+\sum_{i=1}^{n} \beta_{i}N_{i}^{2}+\sum_{i=1}^{n} \alpha_{i}N_{i}^{2}RT+\varepsilon$ (Eq.5)

using (Eq.3).

To test whether correlational effects of nutrient intake on response variables differed, the *SS_r_* from the reduced model:

 (Eq.6)

was compared to the *SS_c_* of the complete model:

**** (Eq.7)

using (Eq.3).

In summary, the comparison of model (Eq.1) versus (Eq.2), (Eq.4) versus (Eq.5), and (Eq.6) versus (Eq.7) provides a test for the overall significance of the interaction between response type and the linear, quadratic and correlational effects of nutrient intake, respectively. Therefore, significant differences in these model comparisons (as detected with a partial *F*-test) demonstrate that the linear, quadratic and/or correlational effects of nutrient intake on the response variables differ, respectively. We also inspected the interaction of individual nutrients with the response variable terms from the full model (Eq.7) to determine which of the nutrients were responsible for the significance of the overall partial *F*-test.

**References**

Bowerman BL, O’Connell RT (1990) *Linear Statistical Models: An Applied Approach*. Duxbury Press, Belmont.

Chenoweth SF, Blows MW (2005) Contrasting mutual sexual selection on homologous signal traits in *Drosophila serrata*. *American Naturalist* 165: 281-289.

Draper NR, John JA (1988) Response-surface designs for quantitative and qualitative variables. *Technometrics* 30: 423-428.

**Text S3. Calculating the angle between linear nutritional vectors and their 95% CI.**

We calculated the angle between two linear nutritional vectors as:

$\theta= \cos^{-1} \left( \frac{a \cdot b}{\|a\|\|b\|} \right)$

where *a* is the linear effects of P and C intake in the first response variable being compared, *b* is the linear effects of these nutrients for the second response variable, $\left\| a \right\|= \sqrt{a \cdot a}$ and $\left\| b \right\|= \sqrt{b \cdot b}$. When ***θ*** = 0°, the vectors are perfectly aligned and the optima for the two response variables are located in the same region in nutrient space, whereas ***θ*** = 180° represents the maximum possible divergence between the nutritional vectors. To determine the significance of ***θ***, we estimated the 95% credible interval using a Bayesian approach implemented in the ‘MCMCglmm’ package of R. For each response variable being compared we ran the following linear model:

$$R=\beta_{1}P+\beta_{2}C+\varepsilon$$

where *R* is the response variable and ***β*_1_** and ***β*_2_** are the nutritional gradients for P and C intake, respectively. For each linear model, we used 400,000 Markov chain iterations with a burn-in of 20,000, a thinning interval of 25 and a relatively uninformative prior (V = 1, nu = 0.02) to create a posterior distribution of ***β***. We used these distributions in the above trigonometry equation to generate 15,200 values for ***θ***. The median of these values were used as our point estimate of ***θ*** and the 2.5% and 97.5% quantiles used as our estimate of the 95% CIs.

The associated R code for this routine is provided below. We provide the code for the comparison of the male sex pheromone (2MT) and gestation time (GT) in females.

library(MCMCglmm)

# read in nutritional data

angle.data<-read.table("Male.txt",h=T)

attach(angle.data)

str(angle.data)

prior<-list(R=list(V=1,nu=0.02))

# str(angle.data) should give 2 columns for dependent variable e.g dietary intake (P and C)

# Bayesian linear regression to estimate beta for each variable, produces

# posterior distribution based on 15200 estimates of each parameter:

angle.model.2MT<-MCMCglmm(2MT~P+C-1,data=angle.data1,prior = prior,nitt=400000,burnin=20000,thin=25)

summary(angle.model.2MT)

# and again for the female reproductive trait (this needs to be done all over again because it is a separate data set)

library(MCMCglmm)

angle.data2<-read.table("Female.txt",h=T)

attach(angle.data2)

str(angle.data2)

prior<-list(R=list(V=1,nu=0.02))

# str(angle.data)

angle.model.GT<-MCMCglmm(GT~P+C-1,data=angle.data2,nitt=400000,burnin=20000,thin=25)

summary(angle.model.GT)

angles<-numeric(15200)

# creates an empty vector the same length as the posterior distribution, in which angle estimates for each row of the posterior

# distribution will be stored as follows:

for(i in 1:15200){

b.2MT<- angle.model.2MT$Sol[i,1:2]

b.GT<- angle.model.GT$Sol[i,1:2]

# creates a vector of beta estimates for each variable for each row of the posterior distribution (and the loop runs through all rows)

angles[i]<- acos((t(b.GT) %*% b.2MT) / ((sqrt(t(b.GT) %*% b.GT)) * (sqrt(t(b.2MT) %*% b.2MT)))) * (180/pi) }

# calculates the angles between 2MT and GT beta's for each row of the posterior distribution

summary(angles)

# provides the mean, median, 1^st^ and 3^rd^ quantiles for the angles produced

HPDinterval(as.mcmc(angles))

# provides the 95% credible interval for the angles produced

**Figure S2.** The mean (±SE) daily amount of diet consumed and the associated intake of protein (P) and carbohydrates (C) in each of the diet pairs by male and female *Nauphoeta cinerea*. (A & B) The daily consumption of each diet by male and female cockroaches, respectively. Grey bars represent the high C diet in the pair and white bars represent the high protein diet in the pair. For each diet pair, paired *t*-test showed that both males and females consumed more of the C rich diet in each pair, irrespective of the total nutrient content of the diet (*males*: Diet pair 1: *t*_19_ = 31.139, *P* = 0.0001; Diet pair 2: *t*_19_ = 17.897, *P* = 0.0001; Diet pair 3: *t*_19_ = 20.590, *P* = 0.0001; Diet pair 4: *t*_19_ = 14.650, *P* = 0.0001; *females*: Diet pair 1: *t*_19_ = 26.567, *P* = 0.0001; Diet pair 2: *t*_19_ = 21.293, *P* = 0.0001; Diet pair 3: *t*_19_ = 17.626, *P* = 0.0001; Diet pair 4: *t*_19_ = 25.955, *P* = 0.0001). (C & D) The difference in the observed P (white bar) and C (grey bar) intake from the expected intake if males and females fed at random on the two diets in a pair, respectively. For each diet pair, both males and females consumed significantly more C than expected had they fed randomly (*males*: Diet pair 1: *t*_19_ = 31.138, *P* = 0.0001; Diet pair 2: *t*_19_ = 17.897, *P* = 0.0001; Diet pair 3: *t*_19_ = 20.590, *P* = 0.0001; Diet pair 4: *t*_19_ = 14.650, *P* = 0.0001; *females*: Diet pair 1; *t*_19_ = 26.576, *P* = 0.0001; Diet pair 2: *t*_19_ = 21.393, *P* = 0.0001; Diet pair 3: *t*_19_ = 17.627, *P* = 0.0001; Diet pair 4: *t*_19_ = 25.955; *P* = 0.0001) and significantly less P than expected (*males*: Diet pair 1: *t*_19_ = 31.138, *P* = 0.0001; Diet pair 2: *t*_19_ = 17.897, *P* = 0.0001; Diet pair 3: *t*_19_ = 20.590, *P* = 0.0001; Diet pair 4: *t*_19_ = 14.650, *P* = 0.0001; *females*: Diet pair 1: *t*_19_ = 26.576, *P* = 0.0001; Diet pair 2: *t*_19_ = 21.392, *P* = 0.0001; Diet pair 3: *t*_19_ = 17.627, *P* = 0.0001; Diet pair 4: *t*_19_ = 25.955, *P* = 0.0001).

**Table S2.** *F* ratio tests comparing the Coefficient of Variation (*CV*) for reproductive traits in (A) males (3H2B, 2MT and 4E2M) and (B) females (clutch size, gestation time) when given the opportunity to choose between alternate diets in one of four different diet pairs versus when cockroaches were exclusively fed only one of the diets in each pair. In each diet pair, HP represents the high protein diet (diet 2 or 4, see Table A1) and HC represents the high carbohydrate diet (diet 22 or 24, see Table A1).

|  |  | ***CV*** | | | |  | ***F* ratio test** | |  |  | |  |  | |
| --- | --- | --- | --- | --- | --- | --- | --- | --- | --- | --- | --- | --- | --- | --- |
| 1. **Males** |  | **Choice** |  | **No-choice** | |  | ***F*_9,19_** | |  | ***P* value** | |  | **% reduction** | |
| Diet pair | Trait |  |  | HP | HC |  | HP | HC |  | HP | HC |  | HP | HC |
| 1 | 3H2B | 0.202 |  | 0.612 | 0.616 |  | 3.027 | 3.045 |  | 0.020 | 0.020 |  | -66.962 | -67.163 |
|  | 2MT | 0.196 |  | 0.608 | 0.620 |  | 3.105 | 3.165 |  | 0.018 | 0.017 |  | -67.791 | -68.403 |
|  | 4E2M | 0.202 |  | 0.608 | 0.609 |  | 3.012 | 3.018 |  | 0.021 | 0.020 |  | -66.804 | -66.862 |
| 2 | 3H2B | 0.180 |  | 0.612 | 0.632 |  | 3.402 | 3.515 |  | 0.012 | 0.010 |  | -70.609 | -71.551 |
|  | 2MT | 0.189 |  | 0.608 | 0.634 |  | 3.220 | 3.358 |  | 0.015 | 0.013 |  | -68.944 | -70.220 |
|  | 4E2M | 0.205 |  | 0.608 | 0.629 |  | 2.959 | 3.066 |  | 0.022 | 0.019 |  | -66.209 | -67.379 |
| 3 | 3H2B | 0.191 |  | 0.588 | 0.616 |  | 3.084 | 3.230 |  | 0.019 | 0.015 |  | -67.575 | -69.036 |
|  | 2MT | 0.175 |  | 0.572 | 0.620 |  | 3.278 | 3.552 |  | 0.014 | 0.010 |  | -69.498 | -71.850 |
|  | 4E2M | 0.202 |  | 0.610 | 0.609 |  | 3.017 | 3.012 |  | 0.020 | 0.021 |  | -66.850 | -66.802 |
| 4 | 3H2B | 0.200 |  | 0.588 | 0.632 |  | 2.935 | 3.156 |  | 0.023 | 0.017 |  | -65.930 | -68.315 |
|  | 2MT | 0.155 |  | 0.572 | 0.634 |  | 3.693 | 4.094 |  | 0.008 | 0.005 |  | -72.921 | -75.572 |
|  | 4E2M | 0.193 |  | 0.610 | 0.629 |  | 3.163 | 3.266 |  | 0.017 | 0.014 |  | -68.383 | -69.380 |
| 1. **Females** |  |  |  |  |  |  |  |  |  |  |  |  |  |  |
| 1 | CS | 0.027 |  | 0.101 | 0.097 |  | 3.712 | 3.556 |  | 0.008 | 0.010 |  | -73.063 | -71.880 |
|  | GT | 0.052 |  | 0.170 | 0.158 |  | 3.287 | 3.067 |  | 0.014 | 0.019 |  | -69.578 | -67.398 |
| 2 | CS | 0.023 |  | 0.101 | 0.076 |  | 4.440 | 3.341 |  | 0.003 | 0.013 |  | -77.476 | -70.065 |
|  | GT | 0.049 |  | 0.170 | 0.146 |  | 3.455 | 2.975 |  | 0.011 | 0.022 |  | -71.058 | -66.389 |
| 3 | CS | 0.032 |  | 0.097 | 0.097 |  | 3.038 | 3.059 |  | 0.020 | 0.019 |  | -67.083 | -67.313 |
|  | GT | 0.049 |  | 0.155 | 0.158 |  | 3.154 | 3.222 |  | 0.017 | 0.015 |  | -68.299 | -68.965 |
| 4 | CS | 0.021 |  | 0.097 | 0.076 |  | 4.563 | 3.610 |  | 0.003 | 0.009 |  | -78.085 | -72.296 |
|  | GT | 0.043 |  | 0.155 | 0.146 |  | 3.609 | 3.402 |  | 0.009 | 0.012 |  | -72.292 | -70.603 |

**Figure S3.** The mean (±SE) reproductive traits in male and female *N. cinerea* across the 24 different artificial diets used in our no-choice experiment. Bars with different letters are significant different after post-hoc analysis. The red, dashed line is the mean of the reproductive traits taken across diet choice pairs in our choice experiment.
